# X-shaped structure of bacterial heterotetrameric tRNA synthetase suggests cryptic prokaryote functions and a rationale for synthetase classifications

**DOI:** 10.1093/nar/gkab707

**Published:** 2021-08-14

**Authors:** Yingchen Ju, Lu Han, Bingyi Chen, Zhiteng Luo, Qiong Gu, Jun Xu, Xiang-Lei Yang, Paul Schimmel, Huihao Zhou

**Affiliations:** Guangdong Provincial Key Laboratory of Chiral Molecule and Drug Discovery, School of Pharmaceutical Sciences, Sun Yat-sen University, Guangzhou 510006, China; Research Center for Drug Discovery, School of Pharmaceutical Sciences, Sun Yat-sen University, Guangzhou 510006, China; Guangdong Provincial Key Laboratory of Chiral Molecule and Drug Discovery, School of Pharmaceutical Sciences, Sun Yat-sen University, Guangzhou 510006, China; Research Center for Drug Discovery, School of Pharmaceutical Sciences, Sun Yat-sen University, Guangzhou 510006, China; Guangdong Provincial Key Laboratory of Chiral Molecule and Drug Discovery, School of Pharmaceutical Sciences, Sun Yat-sen University, Guangzhou 510006, China; Research Center for Drug Discovery, School of Pharmaceutical Sciences, Sun Yat-sen University, Guangzhou 510006, China; Guangdong Provincial Key Laboratory of Chiral Molecule and Drug Discovery, School of Pharmaceutical Sciences, Sun Yat-sen University, Guangzhou 510006, China; Research Center for Drug Discovery, School of Pharmaceutical Sciences, Sun Yat-sen University, Guangzhou 510006, China; Research Center for Drug Discovery, School of Pharmaceutical Sciences, Sun Yat-sen University, Guangzhou 510006, China; Research Center for Drug Discovery, School of Pharmaceutical Sciences, Sun Yat-sen University, Guangzhou 510006, China; Department of Molecular Medicine, The Scripps Research Institute, La Jolla, CA 92037, USA; Department of Molecular Medicine, The Scripps Research Institute, La Jolla, CA 92037, USA; Department of Molecular Medicine, The Scripps Research Institute, Jupiter, FL 33458, USA; Guangdong Provincial Key Laboratory of Chiral Molecule and Drug Discovery, School of Pharmaceutical Sciences, Sun Yat-sen University, Guangzhou 510006, China; Research Center for Drug Discovery, School of Pharmaceutical Sciences, Sun Yat-sen University, Guangzhou 510006, China

## Abstract

AaRSs (aminoacyl-tRNA synthetases) group into two ten-member classes throughout evolution, with unique active site architectures defining each class. Most are monomers or homodimers but, for no apparent reason, many bacterial GlyRSs are heterotetramers consisting of two catalytic α-subunits and two tRNA-binding β-subunits. The heterotetrameric GlyRS from *Escherichia coli* (*Ec*GlyRS) was historically tested whether its α- and β-polypeptides, which are encoded by a single mRNA with a gap of three in-frame codons, are replaceable by a single chain. Here, an unprecedented X-shaped structure of *Ec*GlyRS shows wide separation of the abutting chain termini seen in the coding sequences, suggesting strong pressure to avoid a single polypeptide format. The structure of the five-domain β-subunit is unique across all aaRSs in current databases, and structural analyses suggest these domains play different functions on α-subunit binding, ATP coordination and tRNA recognition. Moreover, the X-shaped architecture of *Ec*GlyRS largely fits with a model for how two classes of tRNA synthetases arose, according to whether enzymes from opposite classes can simultaneously co-dock onto separate faces of the same tRNA acceptor stem. While heterotetrameric GlyRS remains the last structurally uncharacterized member of aaRSs, our study contributes to a better understanding of this ancient and essential enzyme family.

## INTRODUCTION

AaRSs establish the rules of the genetic code by covalently linking an amino acid to its cognate tRNA, and therefore play irreplaceable roles in protein synthesis (1,2). Corresponding to the twenty amino acids used in protein synthesis, cellular lives usually have a full set of twenty aaRSs. These aaRSs evolved from two different ancestral active site domains and are thus classified as class I or class II (3,4). Class I enzymes are either monomers or dimers, that are formed from a Rossmann fold that binds the tRNA acceptor stem from the minor groove side, whereas class II aaRSs are either dimers or tetramers with a core active site of antiparallel β strands that binds to the major groove side of the acceptor stem (5). It is believed that a full set of twenty aaRSs emerged before the tree of life diverged from the last common ancestor (LUCA) into three domains (6). After that, aaRSs underwent extensive horizontal gene transfer during evolution which led to the insertion of new sequences or domains (especially in eukaryotic aaRSs) (7,8), endowing the aaRSs with better efficiency, fidelity and regulation and, even more interestingly, abundant non-canonical functions beyond protein translation (9,10). Despite the significant diversity of protein sequences and domain organizations of aaRSs from different organisms, it is clear that, for a specific amino acid, the aaRSs from three kingdoms share a common ancestor and catalytic mechanism in most cases (8).

For no apparent reason, two types of glycyl-tRNA synthetase (GlyRS) exist in different organisms. Eukaryotes, archaea and some bacteria have a homodimeric GlyRS belonging to typical class II aaRSs (11), while other bacteria exclusively utilize a heterotetrameric GlyRS with two α-subunits and two β-subunits (termed (αβ)_2_ as explained later) to produce glycyl-tRNA^Gly^ (12,13). The α-subunits from (αβ)_2_*Campylobacter jejuni* GlyRS (*Cj*GlyRS) and *Aquifex aeolicus* GlyRS (*Aa*GlyRS) have been crystallized as α_2_ homodimers (14,15). Although the α-subunit of (αβ)_2_ GlyRS encodes the core catalytic domain with all three signature motifs of class II aaRSs (16), its larger β-subunit is not similar in sequence to α_2_ GlyRS or to other class II aaRSs (17). The α-subunit alone possesses no or extremely low activity even for amino acid activation, the first step of catalysis, indicating that both α- and β-subunits are indispensable for the aminoacylation of tRNA^Gly^ (18). A later study revealed that the N-terminal part of the β-subunit is necessary for adenylate synthesis, while the C-terminal part harbors a tRNA binding domain (19). The central part of the β-subunit was predicted to be a hydrolase domain (HD) that is frequently observed in metal-dependent phosphohydrolases, but it does not have the requisite histidine-aspartate catalytic doublet, which leaves its function unclear (17).

The α_2_ and (αβ)_2_ GlyRSs are so unrelated in sequence, domain organization and strategies for amino acid recognition that they ostensibly originated from different ancestors (20), which thus challenges the models for the evolution of the aaRS family (21). Supporting this point, the distribution of GlyRS types in different bacteria also does not correlate with the evolutionary emergence of these bacteria (22). From a practical point of view, its unique features provide a larger chemical space to seek bacteria-specific inhibitors that do not cross-react with human GlyRS and, consequently, lessen the problem of toxicities in the drive to find new antibiotics to combat emerging antibiotic resistances.

To date, the singular (αβ)_2_ GlyRS overall structure remains undetermined. Herein, we report a crystal structure of *Escherichia coli* GlyRS (*Ec*GlyRS) with a resolution of 2.68 Å that adopts an unprecedented X-shaped architecture. The structure reveals five domains in the β-subunit. While some of the domains facilitate the formation of the intact active pocket by the α-subunit, some domains may contribute to tRNA binding by showing significant structural similarity to tRNA CCA-adding enzymes and a tRNA recognition domain in alanyl-tRNA synthetase (AlaRS). Moreover, the evolutionary strategy of this peculiar family of GlyRSs appears to select for a large separation of distinct parts of what normally would be a single polypeptide chain, which enables an X-shaped architecture as shown here. Despite this unprecedented structural format, the resulting enzyme appears to conform to a tRNA docking scheme that pairs two specific synthetases on one tRNA, thereby reinforcing an evolutionary model that provides a rationale for the two classes of synthetases and their subclasses.

## MATERIALS AND METHODS

### Protein expression and purification of *Ec*GlyRS

The genes *glyQ* and *glyS* encoding the α- and β-subunits of *Ec*GlyRS were amplified from the genomic DNA of *E. coli* K-12 by PCR. The full-length α-subunit (res. 1–303) was inserted into the pET20b vector (Novagen) using the NdeI and XhoI sites. For crystallization, the β-subunit fragment (res. 1–575) was subcloned into the pET29b vector (Novagen) with a C-terminal 6 × His tag using the NdeI and XhoI sites. BL21(DE3) *E. coli* cells (Novagen) carrying both plasmids were grown to OD_600_ = 0.6 in LB medium supplemented with 100 μg/ml ampicillin and 50 μg/ml kanamycin. The overexpression of the β-subunit-truncated *Ec*GlyRS (named *Ec*GlyRS575) protein was induced by adding 0.1 mM IPTG and was shaken at 220 rpm at 25°C for 16 h. The cells were harvested by centrifugation at 4000 rpm at 4°C for 30 min. Cells were resuspended and sonicated with ice-cold lysis buffer (200 mM NaCl, 50 mM Tris pH 8.0, 20 mM imidazole). Cell lysates were centrifuged at 18 000 rpm for 30 min, and the supernatant was loaded onto a Ni-NTA column (Qiagen) pre-equilibrated with lysis buffer. The Ni-NTA column was washed with 20 column volumes of lysis buffer to remove impurities, and the *Ec*GlyRS575 protein was eluted with 50 ml of elution buffer (200 mM NaCl, 50 mM Tris–HCl, pH 8.0, 200 mM imidazole). The protein in the elution fraction was concentrated to 15–20 mg/ml using a 50 kDa Ultra-15 centrifugal filter device (Millipore) and was then further purified by size-exclusion chromatography (Cytiva, HiLoad Superdex 200 pg) with SEC buffer (100 mM NaCl, 10 mM Tris–HCl, pH 8.0). The purified *Ec*GlyRS575 protein was desalted and concentrated in a storage buffer (50 mM NaCl, 5 mM Tris–HCl, pH 8.0) and frozen at –80°C before use.

The selenomethionine-substituted *Ec*GlyRS575 (SeMet-*Ec*GlyRS575) protein was expressed with *E. coli* strain B834 (DE3) (Novagen) using M9 medium supplemented with selenomethione as previously described (23), and then purified the same as the native *Ec*GlyRS575 protein.

The full-length β-subunit (res. 1–689) was also inserted into the pET29b vector (Novagen) the same as the β-subunit fragment (res. 1–575). All of the site-directed mutations of *Ec*GlyRS-FL were constructed using the QuickChange Directed Mutagenesis Kit (Agilent Technologies) according to the manufacturer's instructions, while the plasmid for expressing the ΔB2 deletion protein (res. 66–124 was replaced by a -GSGS- linker) was generated through a homologous recombination method. The expression and purification of these *Ec*GlyRS variants were the same as those of *Ec*GlyRS575.

### Crystallography

SeMet-*Ec*GlyRS575 protein (30 mg/ml) was mixed with 2 mM adenylyl-imidodiphosphate (AMP-PNP, Sigma-Aldrich) and 5 mM glycine (Sigma-Aldrich) and incubated on ice for 30 min. Crystals were grown using the sitting-drop vapor-diffusion method, in which 1 μl of protein solution was mixed with an equal volume of reservoir solution and then equilibrated against 70 μl of reservoir solution at 18°C. Large crystals appeared after 3 days in the wells using the reservoir solution consisting of 0.4 M magnesium acetate, 0.1 M HEPES pH 6.8, 2% (v/v) PEG 3350, 2% (v/v) PEG 5000 MME, 2% (v/v) PEG 4000, 2% (v/v) PEG 2000 and 10% ethylene glycol. Single-wavelength anomalous diffraction (SAD) data were collected at 0.9792 Å using a single crystal at 100 K on beamline BL19U1 of the National Center for Protein Sciences Shanghai (NCPSS) and the Shanghai Synchrotron Radiation Facility (SSRF). The oscillation angle is 1° for each frame, and the whole data set contains 360 frames.

The diffraction data were processed and scaled with HKL3000 (24). The initial phases were calculated and the model was automatically built using the Crank2 pipeline in CCP4 (25). The structure model was further refined in Coot (26) and Refmac5 (27). The final structure model was refined to 2.68 Å with *R/R*_free_ = 23.89%/25.29% and achieved good stereochemistry quality as assessed by the program MolProbity (28). The statistics of data collection and structure refinement are listed in Supplementary Table S1. The structure was analyzed in PyMOL (www.pymol.org), which was also used to create figures.

### Measurement of glycine activation activity of *Ec*GlyRS

Glycine activation by *Ec*GlyRS and its variants was detected by employing a coupled-assay as previously described (29,30), with a few modifications. Briefly, in the active site of *Ec*GlyRS, the reactions between an AMP-PNP and a glycine generate a molecule of glycyl-AMP that would be attacked by a pyrophosphate to form glycine and one ATP. Hexokinase consumes one ATP to phosphorylate glucose to glucose 6-phosphate, from which glucose 6-phosphate dehydrogenase then consumes one NADP^+^ to produce one glucose-6-phosphate and one NADPH. Thus, the formation of one glycyl-AMP will finally cause the production of one NADPH in this coupled continuous assay. The experiment was conducted at room temperature in a clear 96-well microplate (Corning). 90 μl of reaction buffer consisting of 50 mM HEPES pH 7.5, 10 mM MgCl_2_, 50 mM KCl, 1 mM dithiothreitol, 5 mM AMP-PNP, 5 mM glycine, 10 mM d-glucose, 2.5 mM sodium pyrophosphate, 0.5 mM NADP^+^, 0.025 U yeast hexokinase and 0.025 U glucose 6-phosphate dehydrogenase was placed in the wells of the microplate. The reactions were started by adding 10 μl of *Ec*GlyRS or its variants (the final concentrations of the enzymes were 200 nM) to each well. The production of NADPH in the coupled-assay was continuously recorded for the first 10 min by monitoring the absorbance at 340 nm using a FlexStation 3 multimode microplate reader (Molecular Devices), and the reactions without adding any *Ec*GlyRS enzyme were used as blank controls. The slope of NADPH formation in the first 10 min (equal to the glycine activation rate) of wild-type *Ec*GlyRS-FL was defined as 100%. The results are from three independent assays, and the error bars are standard deviations (SD).

### RNA transcription

*Escherichia coli* tRNA^Gly^ (anticodon: GCC, tRNAdb ID: tdbD00000838) was produced by *in vitro* transcription using T7 polymerase. The DNA template was generated by PCR using Primer1 (5′-**TAATACGACTCACTATA**GCGGGAATAGCTCAGTTGGTAGAGCACGACCTTGCCAAG-3′) and Primer2 (5′-TGGAGCGGGAAACGAGACTCGAACTCGCGACCCCGACCTTGGCAAGGTCGTGCTC-3′). The underlined nucleotides overlap between two primers, and the nucleotides in bold are T7 promoters. The PCR product of Primer1 and Primer2 was further amplified by PCR using Primer3 (5′-TAATACGACTCACTATAGCGGGAATAGC-3′) and Primer4 (5′-TGGAGCGGGAAACGAGACTCG-3′), and the PCR product was directly used as the DNA template for *in vitro* T7 transcription without any further purification. The T7 transcription reaction contained 40 mM Tris–HCl pH 8.0, 1 mM spermidine, 5 mM DTT, 1% (v/v) Triton X-100, 20 mM MgCl_2_, 50 μg of DNA template, 0.4 mM ATP, 0.4 mM UTP, 0.4 mM GTP and 0.4 mM CTP. 1 ml of the reaction was incubated at 37°C for 3–4 h, and then the transcripts were purified by using denaturing 12% polyacrylamide gel electrophoresis. The concentrations of the purified tRNA products were determined through the UV absorbance at a wavelength of 260 nm.

### ATP consumption assay

The ATP consumption assay was employed to evaluate the aminoacylation activity (including both steps of glycine activation and tRNA charging) of *Ec*GlyRS and its variants. The 100 μl reactions consisted of 50 nM *Ec*GlyRS or its variants, 200 μM ATP, 500 μM glycine, 5 mg/ml *in vitro* transcribed *E. coli* tRNA^Gly^ (anticodon: GCC), 30 mM HEPES pH 7.5, 150 mM NaCl, 30 mM KCl, 40 mM MgCl_2_, 0.1% BSA and 1 mM DTT. Reactions were incubated at room temperature, a 10 μl aliquots of the reactions were moved at different time points (2, 5, 10, 20, 40 min) to a 384-well microplate with 10 μl of Kinase-Glo™ Reagent (Promega) to stop the reaction, and after 10 min incubation, the luminescence (L) was read on a FlexStation 3 multimode microplate reader (Molecular Devices). The concentrations of remaining ATP at each time point were calculated according to the standard curve generated by different concentrations (10, 25, 50, 100, 200 and 400 μM) of ATP (Sigma-Aldrich). Finally, ATP consumption was obtained from the difference between the molar quantities of remaining ATP in each well and initial ATP (2000 pmol). Each reaction group was repeated three times, and the results were expressed as the mean ± SD (*n* = 3). Statistical analyses were performed with GraphPad Prism 7.0 software, and a one phase association equation was used for the curve fitting of the ATP consumption assay.

### Small-angle X-ray scattering (SAXS) analysis

The SAXS data were collected on beamline BL19U2 of the National Center for Protein Sciences Shanghai (NCPSS) and the Shanghai Synchrotron Radiation Facility (SSRF). X-ray scattering was performed at a wavelength of λ = 1.033 nm (12.0 keV). The distance between the sample and detector was 2.626 m, leading the momentum transfer *q* to be recorded in the range of 0.076–3.74 nm^–1^. The scattering profiles were measured using 50 μl of protein solution at 5 mg/ml for *Ec*GlyRS-FL and 8 mg/ml for *Ec*GlyRS575. Both proteins were pre-equilibrated in a SAXS buffer (100 mM NaCl, 50 mM Tris–HCl pH 8.0) and loaded into a fully automated sample system on the BL19U2 beamline. A set of 15 two-dimensional images was recorded for each buffer or sample solution measurement with an exposure time of 1 s per image and was then reduced on-site to one-dimensional scattering profiles using the BioXTAS RAW software package (31).

The SAXS data were analyzed using the ATSAS suite (32). The radius of gyration (*R*_g_) and the scattering intensity *I*(0) were calculated using PRIMUS (33) according to the Guinier approximation at low *q* values in the range of *qR*_g_ < 1.3. The *P*(*r*) values and the maximum dimension of the protein (*D*_max_) were also estimated from the scattering profile using the indirect Fourier transform method implemented in the GNOM program (34). The twenty independent *ab initio* shape envelopes for each protein were calculated using DAMMIF (35) and represented by an ensemble of densely packed beads. The independent models were averaged and superimposed by DAMAVER (36) to generate the final model. The theoretical scattering intensities of the crystal structure or the assembly model were calculated and fitted to the experimental scattering intensity using CRYSOL (37).

### Isothermal titration calorimetry (ITC) assay

To detect the binding of ATP to *Ec*GlyRS-FL and the α-subunit, *Ec*GlyRS-FL (20 μM) or the α-subunit (100 μM) was placed in the sample cell with buffer (20 mM HEPES 7.0, 100 mM NaCl, 2 mM MgCl_2_ and 20% glycerol), 200 mM ATP was loased in the syringe, and then the titration assays were performed at 28°C, with 5 μl for the first injection and 10 μl for the next 19 injections. The interval between two injections was 150 s. The disassociation constants (*K*_d_) were determined by fitting the calorimetric data to a one-site binding model using Origin for ITC software (version 7).

### Size-exclusion chromatographic (SEC) assay

The binding between tRNA^Gly^ and two *Ec*GlyRS truncations (*Ec*GlyRSΔB2 and *Ec*GlyRS575) was analyzed by size-exclusion chromatography. *in vitro* transcribed *E. coli* tRNA^Gly^ (anticodon: GCC, 80 μM) was co-incubated with 20 μM protein at room temperature for 30 min in the solution containing 20 mM Tris–HCl pH 7.0, 100 mM NaCl, 2 mM MgCl_2_, 5 mM glycine, 5 mM AMP and 5% glycerol. The sample was loaded onto a Superdex 200 Increase 10/300 GL column (Cytiva) and the elution profile was monitored at 260 and 280 nm. As a control, 20 μM *Ec*GlyRS-FL was also co-incubated with 80 μM tRNA^Gly^ and the above steps were performed under the same conditions.

## RESULTS

### Overview of the heterotetrameric structure of *Ec*GlyRS

#### Organized as a dimer of two protomers

**Figure 1.**
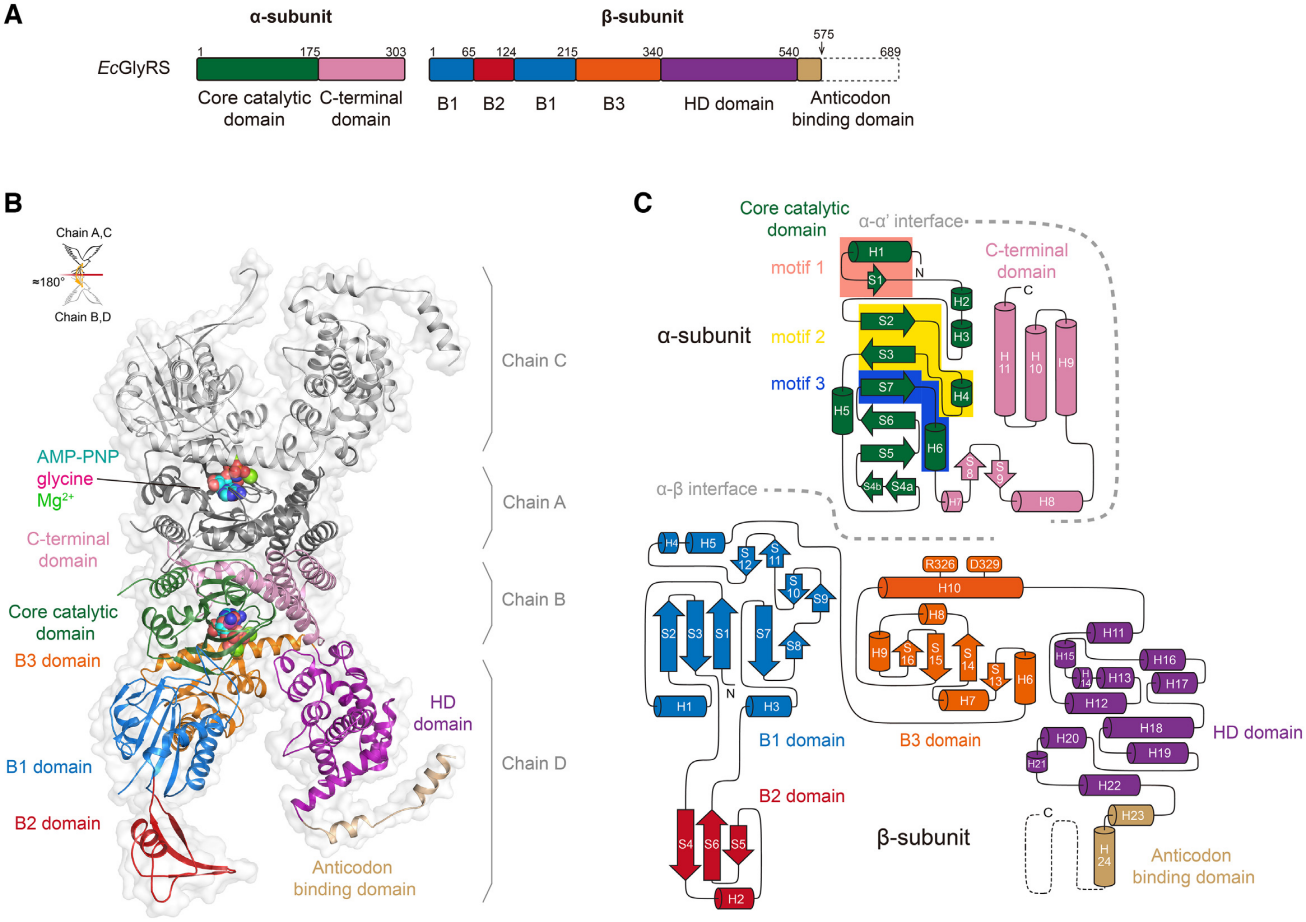
The overall structure of heterotetrameric *Ec*GlyRS. (**A**) The domain organization of *Ec*GlyRS. To facilitate crystallization, the C-terminal part of the anticodon binding domain was truncated, resulting in the construct named *Ec*GlyRS575. (**B**) Cartoon representation of the overall structure of (αβ)_2_ heterotetrameric *Ec*GlyRS575. The protomer consisting of chains B and D are colored as shown in Figure. 1A, while the other protomer (chains A and C) are colored gray. The glycine, Mg^2+^, and ATP analog (AMP-PNP) are shown as sphere models in the two aminoacylation pockets. Two protomers are organized through a noncrystallographic 2-fold axis to form the functional heterotetramer. (**C**) The topology diagram of the α- and β-subunits is colored as depicted in Figure 1A. Arrows represent β-strands, while α- and 3_10_-helices are shown as cylinders. Class II signature motifs 1, 2 and 3 in the α-subunit are highlighted in salmon, yellow and blue, respectively, and the residues Arg326 and Asp329 in the β-subunit B3 domain contributing to ATP binding are labeled.

*Ec*GlyRS (EC 6.1.1.14) is operationally a large protein machine of >2000 residues (with molecular weights of 34.7 kDa for the α-subunit and 76.8 kDa for the β-subunit; Figure 1A). Consistent with earlier work (19), we found that a construct that removed 114 amino acids from the C-terminus of the β-subunit was well-expressed and formed an active (αβ)_2_ heterotetramer. The 114 aa C-terminal deletion construct was named *Ec*GlyRS575 (Figure 1A). In solution, *Ec*GlyRS575 was shown to form a tight heterotetramer (Supplementary Figure S1) and had a SAXS envelope similar to that of full-length *Ec*GlyRS (*Ec*GlyRS-FL) (Supplementary Figure S2). Through reiterative trial and error we obtained high-quality crystals of *Ec*GlyRS575, and a structural model was refined to 2.68 Å (with an *R/R*_free_ factor of 23.9%/25.3%, Supplementary Table S1).

The crystallographic asymmetric unit contains one heterotetramer, and bound glycine and AMP-PNP, a non-hydrolyzable analog of ATP, are observed in both aminoacylation pockets (Figure 1B). *Ec*GlyRS575 presents an unprecedented X-shape overall structure, with the two α-subunits forming a globular dimer at the center and two β-subunits flanking to each side of the α-subunit dimer (Figure 1B). A protomer is formed by one α-subunit plus one β-subunit, and *Ec*GlyRS is a dimer of two protomers that are organized approximately symmetrically around a 2-fold axis (Figure 1B). The subunit organization formula of heterotetrameric GlyRSs is more accurately expressed as (αβ)_2_ than the previously used α_2_β_2_. As analyzed by PISA (38), the interface between two α-subunits buries an area of 2447.6 Å^2^, and the interfaces between α- and β-subunits of α-β protomers are 2087.3 and 2107.8 Å^2^, respectively. Remarkably, no interaction between the two β-subunits, or between the α- and β-subunits from different protomers occurs.

The structures of the two protomers are similar with root mean square deviations (RMSD) of 0.18 Å for the α-subunits (281 superposable Cα atoms) and 0.56 Å for the β-subunits (500 superposable Cα atoms). Thus, unless otherwise indicated, only the structure of the protomer consisting of chain B and D was analyzed due to its better electron density (Supplementary Figure S3A).

#### Core catalytic domain of the α-subunit

Figure 1C shows the topology of *Ec*GlyRS. Each α-subunit is composed of an N-terminal core catalytic domain (β-strands S1-S7 and helices H1-H6), a C-terminal three-helix domain (H9-H11), and a structured linker between them (β-hairpin S8-S9 and helices H7-H8). The core catalytic domain of the α-subunit contains signature motifs 1–3 of class II aaRSs (Figure 1C, Supplementary Figures S3B and S4), which create a large cavity for ATP and glycine binding. The dimer interface between two α-subunits is mainly formed by their class II-defining motif 1 (H1 and S1) and helices H8-H10. Consistent with a previous analysis (14), the interactions between two α-subunits are mostly provided by H-bonds and salt bridges, which is significantly different from the dimerization interface of the catalytic domain of other class II aaRSs where hydrophobic interactions play a major role (39,40).

#### Five domains make up the β-subunit

The β-subunit folds into an arch-like structure with the top of the arch attached to the α-subunit at the side opposite to the α_2_ dimerization interface. The β-subunit can be divided schematically into five domains according to its tertiary structure (Figure 1): the B1 domain (res. 1–65, 125–215), B2 domain (res. 66–124), B3 domain (res. 216–340), HD hydrolase domain (res. 341–540), and anticodon binding domain (ABD, res. 541 to C-terminus). The B1 domain adopts an α-β-α sandwich structure, which is composed of a five-stranded antiparallel β-sheet (S1–S3 and S7–S8) with two α-helices (H1 and H3) packed on one side and two α-helices (H4 and H5) and two short β hairpins (S9–S10 and S11–S12) packed on the other side (Figure 1C). The B2 domain (res. 65–125) is composed of a three-stranded long β-sheet (S4–S6) attached by an α-helix (H2). The B3 domain forms another α–β–α sandwich structure with an antiparallel β-sheet (S13–S16) surrounded by five α-helices (H6-H10). While the B1 and B3 domains form tight contacts with the α-subunit to form the tetrameric GlyRS, the B2 domains are stretched out from the B1 domain. When the two protomers are aligned, the B2 domains are seen to rotate ∼40° (Supplementary Figure S5).

The HD domain consists of twelve α-helices (H11–H22). The C-terminal ABD of the β-subunit was partly truncated, while the remaining residues form two α-helices (H23 and H24) in an extended conformation. Interestingly, crystal packing analysis revealed that these two remaining α-helices of ABD form domain-swapping interactions with the same motif of *Ec*GlyRS form the adjacent asymmetric unit (Supplementary Figure S6). This is believed to be due to artificial interactions in crystal packing, but it well explains why *Ec*GlyRS575 could grow better crystals than *Ec*GlyRS-FL and other variants.

### The β-subunit supports substrate recognition by the α-subunit

#### Capture of ATP and glycine by the α-subunit alone is consistent with its class II active site signature motifs

**Figure 2.**
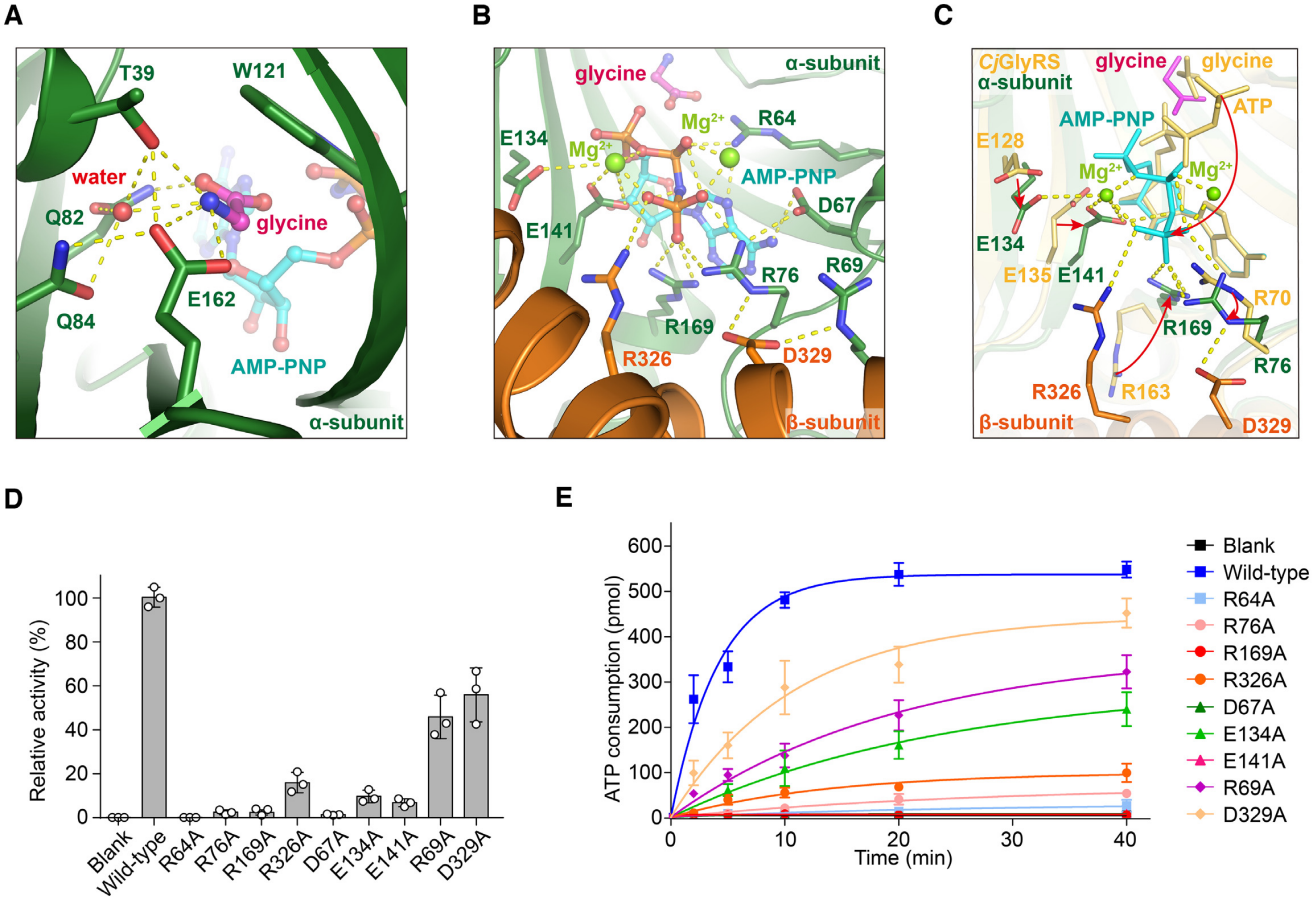
The aminoacylation pocket of *Ec*GlyRS. Glycine (**A**) and AMP-PNP (**B**) binding to the aminoacylation pocket. The bound glycine (magenta) and AMP-PNP (cyan) are shown as ball-and-stick representations, and Mg^2+^ (light green) ions are shown as spheres. The yellow dashed lines indicate the polar contacts. (**C**) Structural comparison of the aminoacylation pockets between *Ec*GlyRS and *Cj*GlyRS (PDB: 3grl, colored yellow), and the major conformation differences in the two structures are indicated by red arrows. (**D**) The activities of glycine adenylation of wild-type *Ec*GlyRS-FL and its variants. The activity of the wild-type enzyme was defined as 100%. (**E**) The aminoacylation activities of *Ec*GlyRS and its variants were detected by measuring ATP consumption. The enzymatic assays here and later were repeated three times, and the error bars indicate the standard deviation (SD).

Previous studies revealed that the active site is located on the α-subunit, which harbors signature motifs 1–3 of class II aaRSs (14). In the *Ec*GlyRS575 structure, glycine and AMP-PNP were observed in both aminoacylation pockets of each protomer (Figure 1B). Glycine was deeply buried in a pocket on the α-subunit that was formed mainly by five residues (Thr39, Gln82, Gln84, Trp121 and Glu162) that are conserved among all (αβ)_2_ GlyRSs (Figure 2A and Supplementary Figure S4). A water molecule contributes to bridging a hydrogen-bond (H-bond) network between these conserved residues and the substrate glycine (Figure 2A). In addition to recognizing glycine, the α-subunit alone can also capture the ATP substrate in an open cleft on the surface (14). The adenine and ribose moieties of ATP insert into the cleft and are mainly formed by signature motifs 2 and 3 (PDB: 3rgl and 3ufg) (14). In contrast, the phosphate groups of ATP were more exposed to solvent and formed very different conformations when binding to the α-subunit alone (Supplementary Figure S7).

#### Support from the β-subunit further stabilizes substrate binding in α-subunit

In our structure of heterotetrameric *Ec*GlyRS, the B3 domain of the β-subunit folds against the α-subunit and partially covers the surface cavity, resulting in the formation of an intact pocket for holding AMP-PNP (Figure 2B and Supplementary Figure S8). The glycine and the adenine and ribose moieties of AMP-PNP are coordinated in this intact pocket with the same manner that they are coordinated by the α-subunit alone (PDB: 3rgl and 3ufg) (14). In contrast, the phosphate groups of AMP-PNP rotate toward the β-subunit (Figure 2C and Supplementary Figure S7). As a result, α-subunit Arg76 and Arg169 form additional charge interactions with the γ-phosphate of AMP-PNP. Asp67, Glu134, and Glu141 also contribute to stabilizing β- and γ- phosphates by coordinating two Mg^2+^ which are necessary for catalysis by class II aaRSs (41,42) (Figure 2B). These interactions and Mg^2+^ were not observed in the structure of the ATP-bound α-subunit of *Cj*GlyRS (Figure 2C). Importantly, Arg326 from the β-subunit B3 domain was observed to form direct charge interactions with the γ-phosphate of AMP-PNP (Figure 2B and C), and Asp329 from the β-subunit B3 domain is involved in stabilizing the conformations of residues Arg76 and Arg69 from the α-subunit which, in turn, contributes to AMP-PNP binding (Figure 2B and C).

The activity of the α-subunit in isolation is extremely low, so the *K*_m_ value for ATP could not be determined. Instead, we tried to measure the disassociation constant (*K*_d_) of ATP binding to the α-subunit alone and to the full enzyme by employing isothermal titration calorimetry (ITC). The results showed that ATP binds to *Ec*GlyRS-FL with a *K*_d_ of about 6 μM, but no significant binding between ATP and the α-subunit alone could be detected (Supplementary Figure S9).

#### Mutational analysis further demonstrates the role of α- and β-subunit cooperation

To further study the recognition of ATP, a set of residues considered to contact the Mg^2+^ or phosphates of AMP-PNP were substituted with alanine, and the resulting variants were measured for their activities for both glycine activation and tRNA^Gly^ aminoacylation (Figure 2D and E). Mutation of Arg64 on the α-subunit, a key residue of motif 2 that contacts an oxygen atom of the β-phosphate, severely decreased the activity of glycine activation (∼985-fold) (Figure 2D). Consistently, the aminoacylation of tRNA^Gly^ by R64A was fully abolished compared to that of wild-type *Ec*GlyRS (Figure 2E). Significant activity decreases were also observed for two other groups of mutations. The first are mutants of three arginine residues proximal to the γ-phosphate of AMP-PNP (Arg76 and Arg169 on the α-subunit and Arg326 on the β-subunit). These mutations decreased glycine activation by ∼47-, ∼123- and ∼15-fold, respectively. The second are mutants of three α-subunit residues—sp67, Glu134 and Glu141—associated with Mg^2+^ binding. These mutations decrease glycine activation by ∼109-, ∼11- and ∼19-fold, respectively (Figure 2D). All of the mutations also significantly decreased the aminoacylation activity of *Ec*GlyRS (Figure 2E).

Thus, although the α-subunit alone is sufficient to bind glycine and ATP in co-crystallization, our structural and biochemical data show that, by directly interacting with the γ-phosphate of ATP and reshaping the conformation of some residues on the α-subunit, the β-subunit facilitates ATP binding and rearranges the conformation of ATP in the aminoacylation pocket.

### The B1-B2 domains of the β-subunit contribute to tRNA binding

#### Searching for structural homologs

To gain more information to understand the evolution and functions of each domain of *Ec*GlyRS, we employed the DALI server to search their structural homologs. For the α-subunit, the best structural homologs in the PDB are α_2_ GlyRSs, followed by the class II AlaRSs AsnRS, LysRS, PheRS, AspRS, etc. (Supplementary Table S2). This result confirms that the α-chain is most similar to other synthetases in class II. The top results also included the ATP phosphoribosyltransferase regulatory subunit (an inactive class II HisRS paralog; PDB: 6r02) and a mitochondrial DNA polymerase accessory subunit (PDB: 1g5h).

For the β-subunit B1-B2 domains, the DALI server identified that the best structural homologs are a class I CCA-adding enzyme (*Af*CCA, an enzyme responsible for the maturation or repair of the functional 3′ end of tRNAs by adding the 3′-essential nucleotides CCA, PDB: 3ovs), a putative transposase (PDB: 2fyx), and the Klenow fragment of DNA polymerase I (PDB: 4yfu) (Supplementary Table S3), but the latter two proteins showed similarity only to the B1 domain (a common fold of the RNA Recognition Motif, RRM) (Supplementary Figure S10).

#### Structural homologs suggested that the B1–B2 domains of the β-subunit facilitate tRNA binding

**Figure 3.**
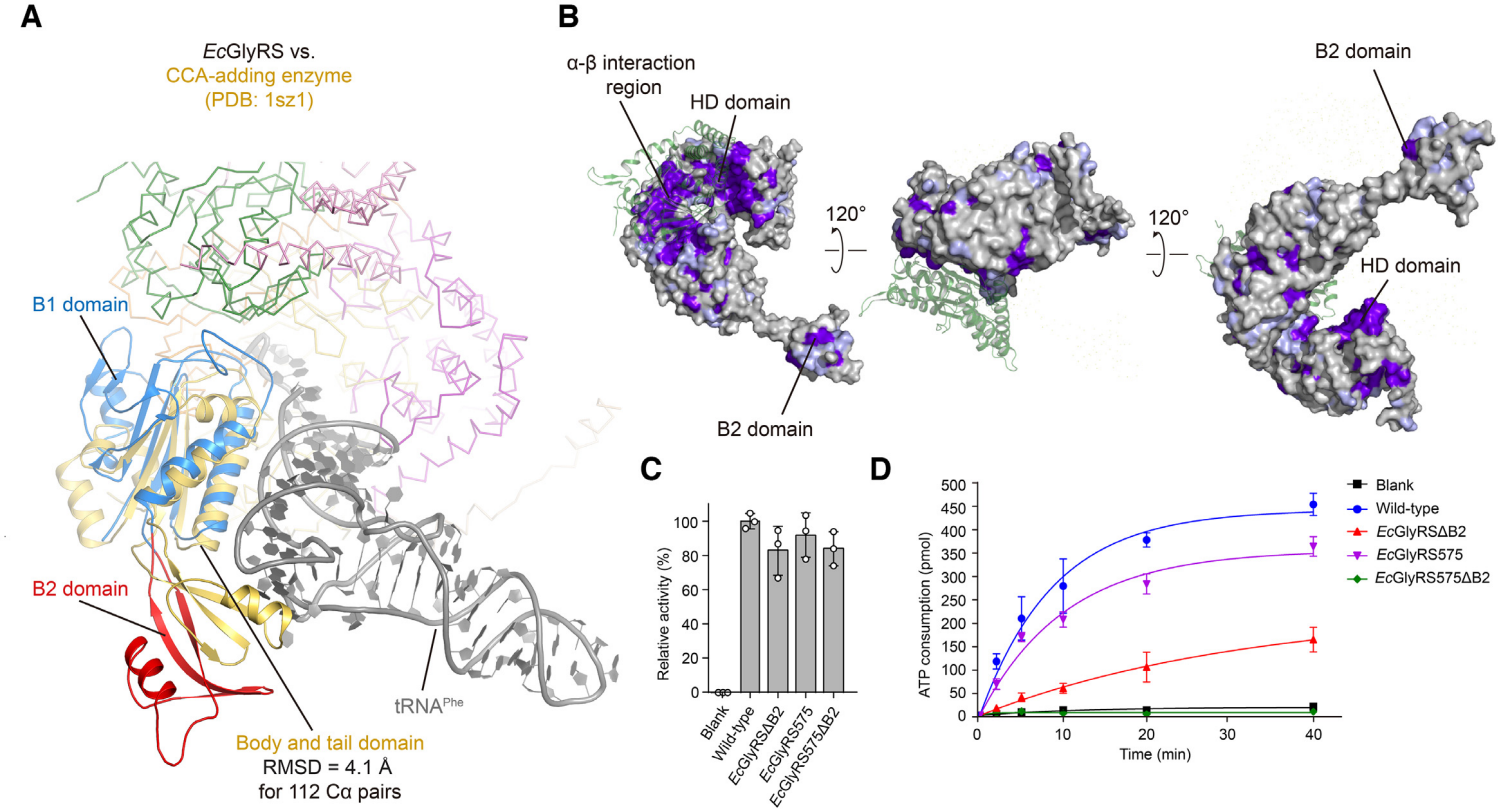
Structural and functional analysis of the B1–B2 domains of the β-subunit. (**A**) Structural superposition of the B1–B2 domains of the β-subunit (colored the same as Figure 1A) and the class I CCA-adding enzyme (*Af*CCA; PDB: 1sz1; yellow) in complex with tRNA^Phe^ (gray). (**B**) The surface conservation profile of the β-subunit of *Ec*GlyRS. The β-subunit is represented as a surface model and colored according to its conservation score, which varies from white (highly variable) to purple (highly conserved). (**C**) Glycine activation of wild-type *Ec*GlyRS and its truncates. (**D**) The aminoacylation activities of *Ec*GlyRS-FL and its truncates.

The body and tail domains of *Af*CCA have significant similarity to the B1–B2 domains of the *Ec*GlyRS β-subunit, respectively (43). When the B1 domain was superimposed to the body domain of *Af*CCA in complex with tRNA^Phe^ (PDB: 1sz1), the B1 domain formed contacts with the acceptor stem of tRNA, and the B2 domain, after a domain shift, could bind to the tRNA elbow region similar to that of the *Af*CCA tail domain (43) (Figure 3A). We analyzed the conserved surface patches on the β-subunit. The β-subunits of twelve GlyRSs with overall sequence identity lower than 35% were aligned (Supplementary Figure S11), and the output was rendered as a surface coloring according to the level of sequence conservation (Figure 3B). In total, three large conserved patches on the β-subunit were identified: a region on B1 and B3 domains that interact with the α-subunit, a region in and around the cavity of the HD domain, and a region on the B2 domain. Importantly, the conserved region on the B2 domain is located on the distal end of B2, which corresponds to the region of *Af*CCA that interacts with the tRNA elbow region. Perhaps of relevance, the elbow was thought to be important for tRNA recognition of some aaRSs (44).

As shown in Figure 3D, removal of the B2 domain (*Ec*GlyRSΔB2) decreased charging activity even more severely than truncation of the ABD (*Ec*GlyRS575). When the B2 and ABD domains were removed simultaneously (*Ec*GlyRS575ΔB2), aminoacylation activity was completely lost (Figure 3D). However, formation of the adenylate intermediate was not strongly impaired in these truncates (Figure 3C), suggesting that the domain truncation mainly affects the tRNA^Gly^-dependent step of aminoacylation rather than glycine activation. Consistently, size-exclusion chromatographic (SEC) assays showed that the *Ec*GlyRS-FL protein can form a small but clear peak of an enzyme-tRNA complex, but the complex peak was not observed for *Ec*GlyRS575 or *Ec*GlyRSΔB2 under the same conditions (Supplementary Figure S12), indicating that truncation of the B2 domain disrupts the *Ec*GlyRS-tRNA^Gly^ interaction.

The B1 domain forms extensive interactions with the α-subunit as well as with the B3 domain of the β-subunit; therefore, the removal of the B1 domain or both B1-B2 domains disrupted the formation of the (αβ)_2_ tetramer (data not shown). Instead, we made single-site mutations on the B1 domain for its positively charged residues that face the tRNA molecule. These residues are mainly located on two loops: the loop between β-strand S1 and α-helix H1 (Lys18 and Arg21) and the loop between H3 and S7 (Lys146, Arg149, Trp150 and Arg159) (Supplementary Figure S13A). While K18A and R21A showed little effect on the tRNA^Gly^ aminoacylation activity of *Ec*GlyRS, mutations of W150A and R159A significantly abolished tRNA^Gly^ aminoacylation (Supplementary Figure S13C). As a control, all mutations were fully active in forming the adenylate intermediate (Supplementary Figure S13B). Thus, the H3–S7 loop of the B1 domain is likely to contribute to tRNA binding.

#### Evidence for structural and functional convergence of evolutionarily distinct GlyRSs

Structural comparisons and mutagenesis analyses suggest the importance of the B1–B2 domains in the productive binding of tRNA^Gly^ to *Ec*GlyRS. More interestingly, the human α_2_ GlyRS (*Hs*GlyRS), although independent in evolution, has an extended and flexible insertion domain (Ins3) that interacts with the elbow region of tRNA^Gly^. Deletion of Ins3 markedly reduced its activity to less than 1% of wild-type activity (45). Remarkably, the *Ec*GlyRS B2 domain and *Hs*GlyRS Ins3 share significant structural similarities (Supplementary Figure S14), and although coming from opposite directions, both approach the elbow region of tRNA^Gly^ (Supplementary Figure S14). This structural and functional convergence in evolution highlights the importance of the elbow region in tRNA^Gly^ binding and aminoacylation.

### Structure and function features of the HD domain

#### Similarities to the HD superfamily of hydrolases

Although the catalytic histidine-aspartate doublet) is changed to Ser393 and Lys394 in *Ec*GlyRS (17), the HD domain (res. 341–540) of the β-subunit showed significant similarity to the HD superfamily of hydrolases, such as the PgpH HD domain (PDB: 4s1b-A, DALI-score: 11.1), the HD-GYP domain cyclic-di-GMP phosphodiesterase (PDB: 4mcw), the *drosophila* MESH1 (PDB: 3nqw) and the *Thermus thermophilus* (P)PPGPP synthetase Spot/RelA (PDB: 6s2t) (Supplementary Table S4). These homologs are related to the accumulation/degradation of endogenous nucleotides. Accordingly, we tested some nucleotides, including c-Di-GMP, cAMP, cGMP, and ppGpp, for binding to the HD domain. They neither increased the *T*_m_ values of the purified HD domain (res. 340–540) in TSA nor affected ATP consumption of *Ec*GlyRS-FL (data not shown).

**Figure 4.**
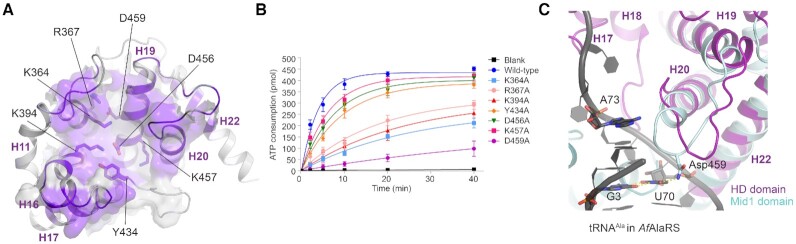
Structural and functional analysis of the HD domain. (**A**) The cavity on the HD domain. The conserved residues in the cavity are colored purple, while other regions are colored white. (**B**) The aminoacylation activities of wild-type *Ec*GlyRS and its variants with single mutations in the HD domain. (**C**) The structural superposition between the HD domain and the Mid1 subdomain of *Archaeoglobus fulgidus* AlaRS (*Af*AlaRS) in complex with tRNA^Ala^ (PDB: 3wqy). Mid 1 (cyan) in the tRNA-recognition domain of *Af*AlaRS and tRNA^Ala^(black) are shown as cartoons. The discriminator base A73 and the G3·U70 pair in tRNA^Ala^ are presented as sticks. The polar contact between Asp459 and U70 is indicated by the yellow dashed line.

Interestingly, the region in and around the large cavity of the HD domain is one of the most conserved surface patches on the β-subunit of (αβ)_2_ GlyRS (Figure 3B). This cavity is mainly formed by residues Lys364, Arg367, Lys394, Tyr434, Asp456, Lys457 and Asp459, and faces the aminoacylation pocket (Figure 4A). Remarkably, although the cavity is more than 30 Å away from the aminoacylation site, mutations of some of the residues did affect the aminoacylation of tRNA^Gly^ (Figure 4B) and did not affect the activation of glycine (Supplementary Figure S15), suggesting a role of the HD domain cavity in the second step of catalysis. It is possible that this cavity functions as an allosteric regulating site. From the practical side, this cavity could be used to target (αβ)_2_ GlyRS-specific inhibitors for antimicrobial drug discovery. The uniqueness of this cavity would diminish possibilities for cross-reaction with the host human cytoplasmic and mitochondrial GlyRSs, which are both of the α_2_ type. It is worth noting that many human pathogens such as *Helicobacter pylori*, *Pseudomonas aeruginosa*, *Streptococcus pneumoniae*, etc. rely on the (αβ)_2_ GlyRS.

#### C-terminal half of the HD domain may play a role in acceptor arm binding

The Mid1 subdomain of *Archaeoglobus fulgidus* AlaRS (*Af*AlaRS, PDB: 3wqy-A) appeared in the top list when we searched for structural homologs of the HD domain, and the C-terminal half (res. 446–540) of the HD domain was able to superpose with the *Af*AlaRS Mid1 subdomain with an RMSD of 3.6 Å for 126 Cα pairs (Supplementary Figure S16). The Mid1 (res. 258–419) and the Mid2 subdomain (res. 420–484) are thought to clamp the major and minor grooves of the acceptor stem and to play an essential role in the recognition of the G3·U70 identity element of tRNA^Ala^ (46). When the Mid1 subdomain of the *Af*AlaRS-tRNA^Ala^ complex was superimposed to the *Ec*GlyRS β-subunit HD domain, the C-terminal half of the HD domain was located close to the acceptor arm of the tRNA molecule (Figure 4C), implying a function of the HD domain in the binding and recognition of tRNA^Gly^. Interestingly, the discriminate U73 of prokaryotic tRNA^Gly^ located beside the N-terminus of helix H19, where amino acid sequences are highly conserved among all heterotetrameric GlyRSs aligned (Supplementary Figure S11).

Notably, C-terminal half of the HD domain was observed to approach the tRNA acceptor arm in a similar manner when the tail and body domains of the *Af*CCA-tRNA^Phe^ complex were superimposed to the *Ec*GlyRS β-subunit B1-B2 domains (Figure 3A), indicating that the *Ec*GlyRS-tRNA binding models suggested by the *Af*CCA-tRNA^Phe^ complex and *Af*AlaRS-tRNA^Ala^ are consistent with each other.

### Structure analysis reveals a large distance between the α-subunit C- and β-subunit N-termini

#### Persistence across bacteria for breaking apart a single chain

**Figure 5.**
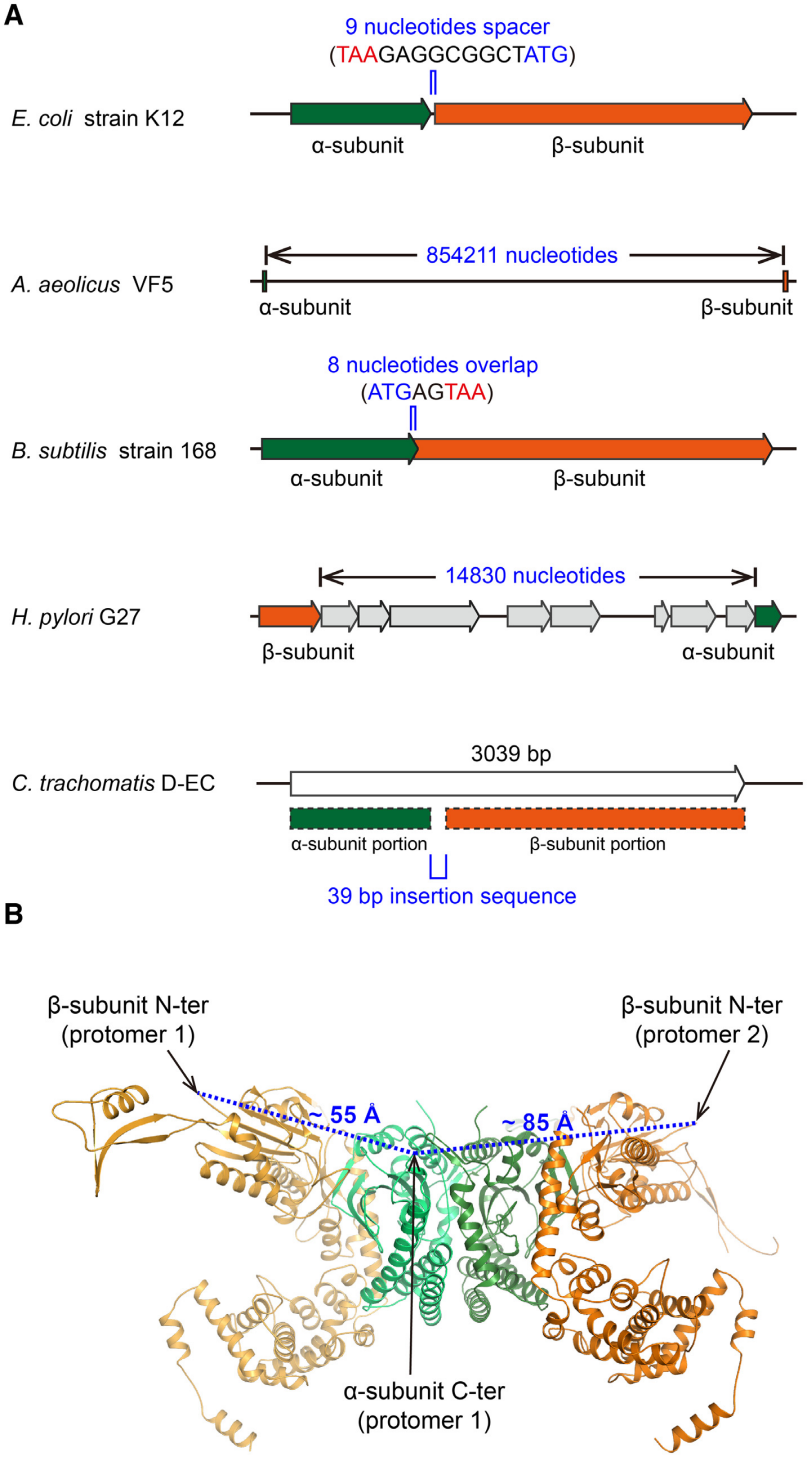
Strong evolutionary pressure separates bacterial-type GlyRSs into two chains. (**A**) Diagrammatic representation of the arrangements of the GlyRS encoding genes found in the genomes of *Escherichia coli* strain K-12 (GenBank: CP009685.1), *Aquifex aeolicus* VF5 (GenBank: AE000657.1), *Bacillus subtilis* strain 168 (GenBank: NC_000964.3), *Helicobacter pylori* G27 (GenBank: CP001173.1) and *Chlamydia trachomatis* D-EC (GenBank: CP002052.1). Green, genes encoding the α-subunit or the α-subunit portion in *Ct*GlyRS; orange, genes encoding the β-subunit or the β-subunit portion in *Ct*GlyRS; gray, genes between the α- and β-subunit in the genome of *Helicobacter pylori* G27. (**B**) The dashed lines (blue) indicate the distance between the C-terminus of the α-subunit and the N-terminus of the β-subunit. The α-subunit (green) and β-subunit (orange) of *Ec*GlyRS in one protomer are shown as cartoons, and the C-terminus of the α-subunit and the N-terminus of the β-subunit are indicated by arrows.

For *Ec*GlyRS, both subunits are encoded in one reading frame in the genome, with an in-frame spacer of 9 nucleotides after a TAA stop interruption at the end of the α-subunit coding region and an ATG start at the beginning of the β-subunit coding region (Figure 5A). Thus, we imaged that in the structure of *Ec*GlyRS, the N-terminus of the β-subunit would be juxtaposed with the C-terminus of the α-subunit. With this in mind, an artificial α–β fusion protein was attempted in an earlier study, but the results were inconclusive (47). Indeed, in our structure, the distance between the C-terminus of the α-subunit and the N-terminus of the β-subunit is ∼55 Å within the same protomer and is even further (∼85 Å) across two protomers (Figure 5B). This observation can explain what appeared to be an unstable fusion protein in an earlier study.

The organization of the α- and β-subunit genes was quite different among different bacteria (48). The distance between the two subunits is 854211 nucleotides in *Aquifex aeolicus* VF5 (Figure 5A). In contrast, in *Bacillus subtillis* strain 168, the C-terminus of the α-subunit coding sequence has an 8-nucleotide overlap with the N-terminus of the β-subunit coding region. In *H. pylori* G27, the β-subunit coding sequence is located upstream of the α-subunit, with several other genes between them. Thus, in all of these examples, the α- and β-subunit coding sequences are separated in each respective genome. Strong evolutionary pressure is likely responsible for this separation.

#### An exception that further supports apparent evolutionary pressure to break the chain

*Chlamydia trachomatis* GlyRS (*Ct*GlyRS) is encoded by a single open reading frame (ORF), and the deduced polypeptide is 1013 residues (21,49) (Figure 5A). The N- and C-terminal regions of the polypeptide show significant similarity to the α- and β-subunits of *Ec*GlyRS, respectively, but not eukaryotic α_2_ GlyRSs, and between the α- and β-subunit coding regions of *Ct*GlyRS is an in-frame linker of 39 nucleotides coding for 13 amino acids. This linker and the tail of the α-subunit are proline-rich and predicted to be flexible, thus they accommodate the separation of subunit termini seen in our structure of *Ec*GlyRS. The fused α- and β-subunits are also observed in other prokaryote-type GlyRSs from chloroplasts of *A. thaliana* and *Phaseolus vulgaris* (21,50). These results further suggest strong evolutionary pressure to separate GlyRSs into two subunits, even when joined together through a linker that facilitates the spacing required for the chain termini to match up with other prokaryote GlyRSs.

Heterotetrameric PheRSs from different bacteria are also organized in either an open or a distal location in the genome (Supplementary Figure S17A). Interestingly, mitochondrial PheRS (*mt*PheRS) is a monomer evolved from bacterial PheRS and is fully active (51). This polypeptide of *mt*PheRS is a fusion of the catalytic module (CAM) of the bacterial α-subunit and the C-terminal domain of the bacterial β-subunit, and many domains involved in the binding of tRNA and in protein quality control have lost during evolution (Supplementary Figure S17B) (51). In contrast, the fused GlyRSs from *Chlamydia trachomatis* and chloroplasts of *A. thaliana* and *Phaseolus vulgaris* retain all the domains of heterotetrameric GlyRS, supporting the proposal that (αβ)_2_ GlyRS is a uniquely ‘obligate’ heterotetramer.

### *Ec*GlyRS supports the subclass-specific pairwise docking of aaRSs on tRNA

#### Specificity of pairing of two aaRSs on one acceptor stem

**Figure 6.**
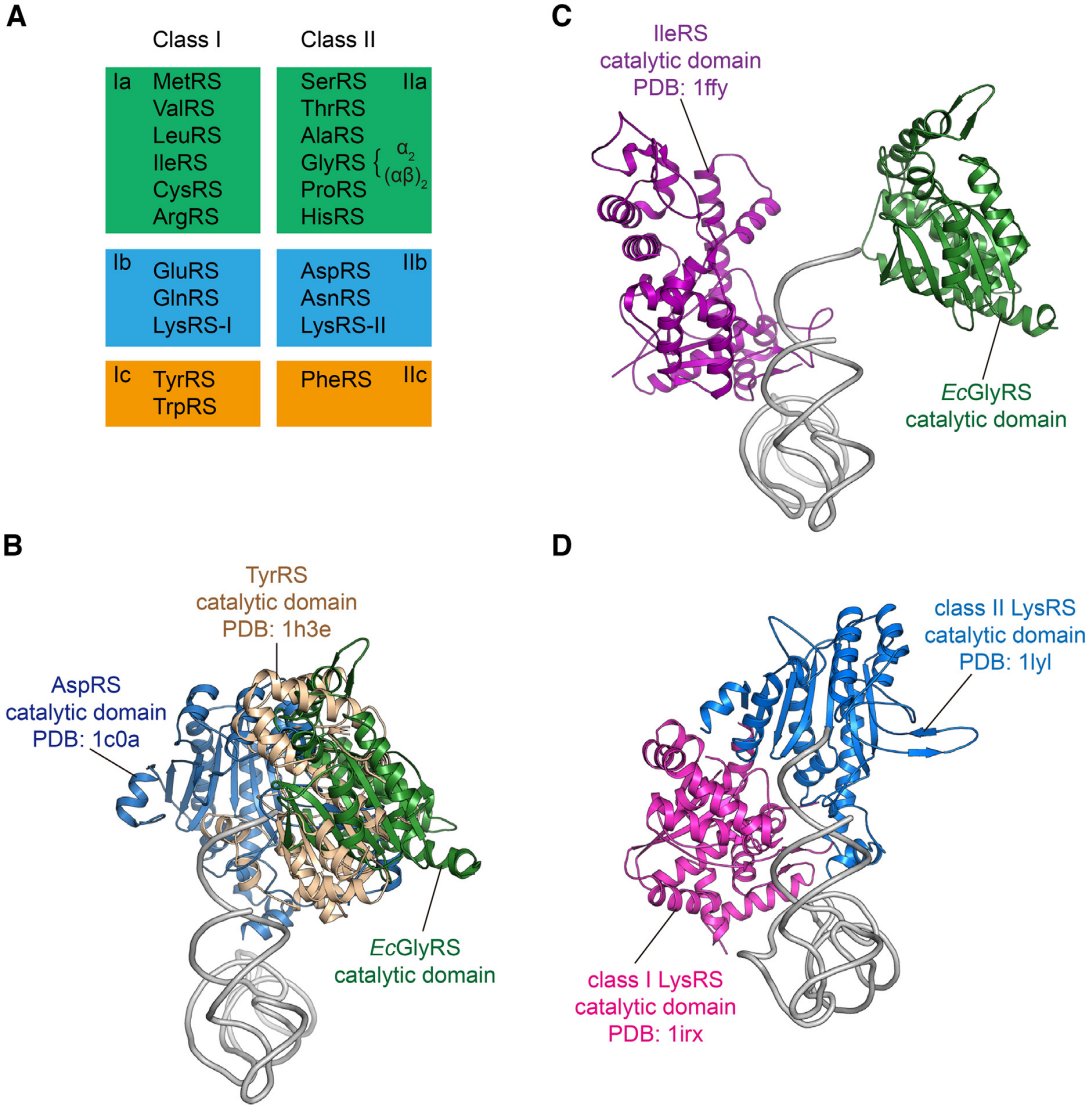
The modeled complexes of aaRS catalytic domains bound simultaneously to a tRNA acceptor stem. (**A**) Classification of class I and II aaRSs. Each enzyme is referred to by the name of its cognate amino acid. The exceptional cases of GlyRS and LysRS where two distinct origins exist for the same enzyme are labeled accordingly. (**B**) The docking result showing that the *Ec*GlyRS and IleRS active sites bound simultaneously to a tRNA acceptor stem. The figure shows the molecules along the axis of the anticodon stem, from the acceptor stem side. (**C**) The docking between the core regions of *Ec*GlyRS, AspRS and TyrRS will result in large steric clashes. (**D**) Class I and II LysRSs, which are the only synthetases known to date that can be of either class I or class II architecture. These two LysRSs can bind to opposite sides of the tRNA acceptor stem without a clash.

The two classes of tRNA synthetases are conserved with rare exceptions through evolution. These two classes, which are based on two distinct architectures for their respective catalytic domains, can be broken down into subclasses (Ia, b, c) and (IIa, b, c) with amino acids of similar size being grouped into the same subclass (Figure 6A) (52). From the early work of Ohno and Rodin and further work by Carter *et al.* on urzymes (53,54), these two classes could in principle arise from opposite strands of early RNA genomes. Co-crystal structures showed that the two classes approach the tRNA acceptor stems from opposite sides (55,56). These observations led to the speculation that primitive tRNA synthetases, among their catalytic roles, served as chaperones to protect the acceptor stems from degradation by nucleases and the high temperatures of early thermophiles (56). To do so, the enzymes need to be paired together in a specific way, to avoid clashing by steric interference. Thus, class Ia enzymes would pair with class IIa enzymes, Ib with IIb, and Ic with IIc. Using structures available at the time, strong support was obtained for this hypothesis.

GlyRS is in class IIa, as part of the six amino acids in that class are matched by rough size with others in class Ia. Of the twelve tRNA synthetases that correspond to those amino acids, as an unusual (αβ)_2_ tRNA synthetase, GlyRS is the only one that is neither a monomer nor a homodimer, and has a completely idiosyncratic structure. Thus, our unusual structure of *Ec*GlyRS presented a singular opportunity to further test the hypothesis concerning the coverage of tRNA acceptor stems.

#### Unusual X-shaped (αβ)_2_ GlyRSs fit the paradigm for specific synthetases pairing on tRNA acceptor stems

As shown in Figure 6B, *Ec*GlyRS fits onto the acceptor stem without a clash with class Ia IleRS or with any of the other class Ia enzymes (Supplementary Figure S18). In contrast, *Ec*GlyRS cannot fit without a steric clash with the class Ib or Ic enzymes (Figure 6C), that is, class I enzymes of a different subclass, suggesting that the (αβ)_2_ GlyRSs belong to class IIa synthetases like the conventional α_2_ GlyRS. Interestingly, however, we found that the tRNA molecules bound from different directions to *Ec*GlyRS and *Hs*GlyRS when their catalytic core domains were aligned (Supplementary Figure S19A). When the tRNA molecules were aligned, the catalytic domains of these two types of GlyRSs were found to bind the acceptor stem of tRNA from different directions (Supplementary Figure S19B). Although the orientations of *Hs*GlyRS and *Ec*GlyRS on the acceptor stem are different, both GlyRSs could dock to tRNA without a clash with its partner class Ia aaRSs(Supplementary Figure S19B).

LysRS is the only aaRS that has examples of class Ib and class IIb architectures. The prediction at the time of the original report was that, when both structures were available, the two enzymes would pair across each other on an acceptor stem, and without a steric clash (55). This expectation was fulfilled (57,58), as shown in Figure 6D.

These results further illustrate how synthetases from opposite subclasses can protect the acceptor stem of the target tRNA. Separately, they are consistent with the idea that the synthetases were ‘born’ in pairs—for example, class Ia enzymes being coded by one strand of a primitive gene and class IIa by the opposite strand (5,53,59). Here, the possibility is raised that the early genetic code was enabled by primitive synthetases that recognized amino acids by size before specificity was developed for side chain fine structure.

## DISCUSSION

### The structure of (αβ)_2_ GlyRS is distinct from that of PheRS

Herein, the first crystal structure of (αβ)_2_ GlyRS from *E. coli* was solved in complex with glycine and the cofactor analog AMP-PNP. Indeed, *Ec*GlyRS shows dramatic structural differences from the classical α_2_ GlyRS in the overall architecture and design of the aminoacylation pockets. Eubacterial and eukaryotic cytoplasmic PheRS is the only other heterotetrameric member in the aaRS family, and also shows an (αβ)_2_ subunit organization pattern (60,61). The overall structure of *Ec*GlyRS is not similar to that of PheRS (60). *Ec*GlyRS forms an X-shape, with only two α-subunits contributing to the oligomerization of the two protomers. No interaction occurs between the two β-subunits or between the α- and β-subunits across two protomers. And, tRNA apparently is engaged by the α- and β-subunits within the same protomer. In contrast, PheRS is shaped as a ‘leatherback turtle’ with large flippers, with each subunit of PheRS contacting more or less with the other three subunits to contribute to tetramerization (60). Thus, although the α- and β-subunits are mainly responsible for catalysis and tRNA recognition, respectively, in both (αβ)_2_ GlyRS and PheRS, the domain organization and the folding of these domains are clearly different in the subunits of the respective synthetases.

### The possible connections of (αβ)_2_ GlyRS with AlaRS and CCA-adding enzymes in evolution

Previous studies revealed that the sequence of the α-subunit of bacterial GlyRS presents some features that resemble AlaRS (e.g. the same sequence insertion in motif 2) (13). Their active sites also share similar overall architectures for recognizing glycine and alanine, the two smallest amino acids in protein synthesis (1,15). This observation suggested that the (αβ)_2_ GlyRS evolved in part from either AlaRS or from an ancestor of AlaRS that was able to aminoacylate both alanine and glycine, while the α_2_ GlyRS evolved from another independent ancestor (15). In our structure, the C-terminal part of the HD domain of *Ec*GlyRS is structurally superposable to Mid1 of AlaRS, a subdomain involved in interacting with the acceptor arm of substrate tRNA^Ala^ (46). This structural feature of *Ec*GlyRS provides additional suggestive evidence, from the side of the β-subunit, that the (αβ)_2_ GlyRS partly evolved in a way related to AlaRS (Supplementary Figure S16 and Figure 4C).

We also showed that mutations in the B1 and B2 domains of the β-subunit affected the tRNA binding of *Ec*GlyRS (Supplementary Figure S13 and Figure 3D). The closest structural homologs of B1-B2 domains in the PDB are the body and tail domains of the class I CCA-adding enzymes, which add CCA to the 3′ ends of all tRNAs for their maturation (43). While the body domain of class I CCA-adding enzymes interacts with the acceptor stem of substrate tRNA, the tail domain is a key binder of the tRNA elbow region (62). Moreover, while the C-terminal part of the *Ec*GlyRS HD domain is structurally superimposable on the Mid1 subdomain of AlaRS, the N-terminal part of the HD domain exhibits significant similarity to the body domain of the class II CCA-adding enzyme (Supplementary Figure S20) (63). With their essential roles in the maturation of tRNAs for protein translation, CCA-adding enzymes are also among the most ancient proteins (62). The potential evolutionary connection between (αβ)_2_ GlyRS and both types of CCA-adding enzymes deserve further investigation.

## DATA AVAILABILITY

Atomic coordinates and structure factors for the reported crystal structure have been deposited with the Protein Data bank under accession number 7EIV.

## Supplementary Material

gkab707_Supplemental_File

## Notes

Present address: Yingchen Ju, Shenzhen Proxybio Therapeutics Co., Ltd, Shenzhen 584000, China.

## References

[1] Rubio Gomez M.A., Ibba M. Aminoacyl-tRNA synthetases. RNA. 2020; 26:910–936. 32303649 10.1261/rna.071720.119 PMC7373986

[2] Ibba M., Soll D. Aminoacyl-tRNA synthesis. Annu. Rev. Biochem. 2000; 69:617–650. 10966471 10.1146/annurev.biochem.69.1.617

[3] Eriani G., Delarue M., Poch O., Gangloff J., Moras D. Partition of tRNA synthetases into two classes based on mutually exclusive sets of sequence motifs. Nature. 1990; 347:203–206. 2203971 10.1038/347203a0

[4] Cusack S., Berthet-Colominas C., Hartlein M., Nassar N., Leberman R. A second class of synthetase structure revealed by X-ray analysis of Escherichia coli seryl-tRNA synthetase at 2.5 Å. Nature. 1990; 347:249–255. 2205803 10.1038/347249a0

[5] Carter C.W. Jr Coding of Class I and II Aminoacyl-tRNA Synthetases. Adv. Exp. Med. Biol. 2017; 966:103–148. 28828732 10.1007/5584_2017_93 PMC5927602

[6] Ribas de Pouplana L. Biology of Aminoacyl-tRNA Synthetases. 2020; 11–37. 10.1016/bs.enz.2020.08.001 33837701

[7] Berthonneau E., Mirande M. A gene fusion event in the evolution of aminoacyl-tRNA synthetases. FEBS Lett. 2000; 470:300–304. 10745085 10.1016/s0014-5793(00)01343-0

[8] Woese C.R., Olsen G.J., Ibba M., Soll D. Aminoacyl-tRNA synthetases, the genetic code, and the evolutionary process. Microbiol. Mol. Biol. Rev. 2000; 64:202–236. 10704480 10.1128/mmbr.64.1.202-236.2000 PMC98992

[9] Kwon N.H., Fox P.L., Kim S. Aminoacyl-tRNA synthetases as therapeutic targets. Nat. Rev. Drug Discov. 2019; 18:629–650. 31073243 10.1038/s41573-019-0026-3

[10] Wei N., Zhang Q., Yang X.L. Neurodegenerative Charcot-Marie-Tooth disease as a case study to decipher novel functions of aminoacyl-tRNA synthetases. J. Biol. Chem. 2019; 294:5321–5339. 30643024 10.1074/jbc.REV118.002955 PMC6462521

[11] Shiba K., Schimmel P., Motegi H., Noda T. Human glycyl-tRNA synthetase. Wide divergence of primary structure from bacterial counterpart and species-specific aminoacylation. J. Biol. Chem. 1994; 269:30049–30055. 7962006

[12] Ostrem D.L., Berg P. Glycyl-tRNA synthetase: an oligomeric protein containing dissimilar subunits. Proc. Natl. Acad. Sci. U.S.A. 1970; 67:1967–1974. 4923123 10.1073/pnas.67.4.1967 PMC283454

[13] Webster T.A., Gibson B.W., Keng T., Biemann K., Schimmel P. Primary structures of both subunits of Escherichia coli glycyl-tRNA synthetase. J. Biol. Chem. 1983; 258:10637–10641. 6309809

[14] Tan K., Zhou M., Zhang R., Anderson W.F., Joachimiak A. The crystal structures of the alpha-subunit of the alpha(2)beta (2) tetrameric Glycyl-tRNA synthetase. J. Struct. Funct. Genomics. 2012; 13:233–239. 23054484 10.1007/s10969-012-9142-6 PMC3691008

[15] Valencia-Sanchez M.I., Rodriguez-Hernandez A., Ferreira R., Santamaria-Suarez H.A., Arciniega M., Dock-Bregeon A.C., Moras D., Beinsteiner B., Mertens H., Svergun D. et al. Structural insights into the polyphyletic origins of glycyl tRNA synthetases. J. Biol. Chem. 2016; 291:14430–14446. 27226617 10.1074/jbc.M116.730382 PMC4938167

[16] Toth M.J., Schimmel P. A mutation in the small (alpha) subunit of glycyl-tRNA synthetase affects amino acid activation and subunit association parameters. J. Biol. Chem. 1990; 265:1005–1009. 2295596

[17] Wolf Y.I., Aravind L., Grishin N.V., Koonin E.V. Evolution of aminoacyl-tRNA synthetases–analysis of unique domain architectures and phylogenetic trees reveals a complex history of horizontal gene transfer events. Genome Res. 1999; 9:689–710. 10447505

[18] Nagel G.M., Cumberledge S., Johnson M.S., Petrella E., Weber B.H. The beta subunit of *E. coli* glycyl-tRNA synthetase plays a major role in tRNA recognition. Nucleic. Acids. Res. 1984; 12:4377–4384. 6374618 10.1093/nar/12.10.4377 PMC318838

[19] Toth M.J., Schimmel P. Deletions in the large (beta) subunit of a hetero-oligomeric aminoacyl-tRNA synthetase. J. Biol. Chem. 1990; 265:1000–1004. 2404005

[20] Freist W., Logan D.T., Gauss D.H. Glycyl-tRNA synthetase. Biol. Chem. Hoppe Seyler. 1996; 377:343–356. 8839980

[21] Ibba M., Francklyn C., Cusack S. The Aminoacyl-tRNA Synthetases. 2005; Georgetown, Texas Landes Bioscience.

[22] Giege R., Springer M. Aminoacyl-tRNA synthetases in the bacterial world. EcoSal Plus. 2016; 7:10.1128/ecosalplus.ESP-0002-2016. PMC11575706 27223819

[23] Ju Y., Tong S., Gao Y., Zhao W., Liu Q., Gu Q., Xu J., Niu L., Teng M., Zhou H. Crystal structure of a membrane-bound l-amino acid deaminase from *Proteus vulgaris*. J. Struct. Biol. 2016; 195:306–315. 27422658 10.1016/j.jsb.2016.07.008

[24] Minor W., Cymborowski M., Otwinowski Z., Chruszcz M. HKL-3000: the integration of data reduction and structure solution–from diffraction images to an initial model in minutes. Acta Crystallogr. D. Biol. Crystallogr. 2006; 62:859–866. 16855301 10.1107/S0907444906019949

[25] Potterton L., Agirre J., Ballard C., Cowtan K., Dodson E., Evans P.R., Jenkins H.T., Keegan R., Krissinel E., Stevenson K. et al. CCP4i2: the new graphical user interface to the CCP4 program suite. Acta Crystallogr D Struct Biol. 2018; 74:68–84. 29533233 10.1107/S2059798317016035 PMC5947771

[26] Emsley P., Lohkamp B., Scott W.G., Cowtan K. Features and development of Coot. Acta Crystallogr. D. Biol. Crystallogr. 2010; 66:486–501. 20383002 10.1107/S0907444910007493 PMC2852313

[27] Murshudov G.N., Skubak P., Lebedev A.A., Pannu N.S., Steiner R.A., Nicholls R.A., Winn M.D., Long F., Vagin A.A. REFMAC5 for the refinement of macromolecular crystal structures. Acta Crystallogr. D. Biol. Crystallogr. 2011; 67:355–367. 21460454 10.1107/S0907444911001314 PMC3069751

[28] Williams C.J., Headd J.J., Moriarty N.W., Prisant M.G., Videau L.L., Deis L.N., Verma V., Keedy D.A., Hintze B.J., Chen V.B. et al. MolProbity: more and better reference data for improved all-atom structure validation. Protein Sci. 2018; 27:293–315. 29067766 10.1002/pro.3330 PMC5734394

[29] Lloyd A.J., Potter N.J., Fishwick C.W., Roper D.I., Dowson C.G. Adenosine tetraphosphoadenosine drives a continuous ATP-release assay for aminoacyl-tRNA synthetases and other adenylate-forming enzymes. ACS Chem. Biol. 2013; 8:2157–2163. 23898887 10.1021/cb400248f

[30] Roy S. A continuous spectrophotometric assay for *Escherichia coli* alanyl-transfer RNA synthetase. Anal. Biochem. 1983; 133:292–295. 6356984 10.1016/0003-2697(83)90086-6

[31] Hopkins J.B., Gillilan R.E., Skou S. BioXTAS RAW: improvements to a free open-source program for small-angle X-ray scattering data reduction and analysis. J. Appl. Crystallogr. 2017; 50:1545–1553. 29021737 10.1107/S1600576717011438 PMC5627684

[32] Franke D., Petoukhov M.V., Konarev P.V., Panjkovich A., Tuukkanen A., Mertens H.D.T., Kikhney A.G., Hajizadeh N.R., Franklin J.M., Jeffries C.M. et al. ATSAS 2.8: a comprehensive data analysis suite for small-angle scattering from macromolecular solutions. J. Appl. Crystallogr. 2017; 50:1212–1225. 28808438 10.1107/S1600576717007786 PMC5541357

[33] Konarev P.V., Volkov V.V., Sokolova A.V., Koch M.H.J., Svergun D.I. PRIMUS: a Windows PC-based system for small-angle scattering data analysis. J. Appl. Crystallogr. 2003; 36:1277–1282.

[34] Svergun D.I. Determination of the regularization parameter in indirect-transform methods using perceptual criteria. J. Appl. Crystallogr. 1992; 25:495–503.

[35] Franke D., Svergun D.I. DAMMIF, a program for rapid ab-initio shape determination in small-angle scattering. J. Appl. Crystallogr. 2009; 42:342–346. 27630371 10.1107/S0021889809000338 PMC5023043

[36] Volkov V.V., Svergun D.I. Uniqueness of *ab initio* shape determination in small-angle scattering. J. Appl. Crystallogr. 2003; 36:860–864. 10.1107/S0021889809000338 PMC5023043 27630371

[37] Svergun D., Barberato C., Koch M.H.J. CRYSOL – a program to evaluate X-ray solution scattering of biological macromolecules from atomic coordinates. J. Appl. Crystallogr. 1995; 28:768–773.

[38] Krissinel E., Henrick K. Inference of macromolecular assemblies from crystalline state. J. Mol. Biol. 2007; 372:774–797. 17681537 10.1016/j.jmb.2007.05.022

[39] Yaremchuk A., Cusack S., Tukalo M. Crystal structure of a eukaryote/archaeon-like protyl-tRNA synthetase and its complex with tRNAPro(CGG). EMBO J. 2000; 19:4745–4758. 10970866 10.1093/emboj/19.17.4745 PMC302085

[40] Arnez J.G., Harris D.C., Mitschler A., Rees B., Francklyn C.S., Moras D. Crystal structure of histidyl-tRNA synthetase from *Escherichia coli* complexed with histidyl-adenylate. EMBO J. 1995; 14:4143–4155. 7556055 10.1002/j.1460-2075.1995.tb00088.x PMC394497

[41] Belrhali H., Yaremchuk A., Tukalo M., Berthet-Colominas C., Rasmussen B., Bosecke P., Diat O., Cusack S. The structural basis for seryl-adenylate and Ap4A synthesis by seryl-tRNA synthetase. Structure. 1995; 3:341–352. 7613865 10.1016/s0969-2126(01)00166-6

[42] Arnez J.G., Augustine J.G., Moras D., Francklyn C.S. The first step of aminoacylation at the atomic level in histidyl-tRNA synthetase. Proc. Natl. Acad. Sci. U.S.A. 1997; 94:7144–7149. 9207058 10.1073/pnas.94.14.7144 PMC23771

[43] Xiong Y., Steitz T.A. Mechanism of transfer RNA maturation by CCA-adding enzyme without using an oligonucleotide template. Nature. 2004; 430:640–645. 15295590 10.1038/nature02711

[44] Zhang J., Ferre-D’Amare A.R. The tRNA elbow in structure, recognition and evolution. Life (Basel). 2016; 6:3. 10.3390/life6010003 PMC4810234 26771646

[45] Deng X., Qin X., Chen L., Jia Q., Zhang Y., Zhang Z., Lei D., Ren G., Zhou Z., Wang Z. et al. Large conformational changes of insertion 3 in human glycyl-tRNA synthetase (hGlyRS) during catalysis. J. Biol. Chem. 2016; 291:5740–5752. 26797133 10.1074/jbc.M115.679126 PMC4786711

[46] Naganuma M., Sekine S., Chong Y.E., Guo M., Yang X.L., Gamper H., Hou Y.M., Schimmel P., Yokoyama S. The selective tRNA aminoacylation mechanism based on a single G*U pair. Nature. 2014; 510:507–511. 24919148 10.1038/nature13440 PMC4323281

[47] Keng T., Webster T.A., Sauer R.T., Schimmel P. Gene for Escherichia coli glycyl-tRNA synthetase has tandem subunit coding regions in the same reading frame. J. Biol. Chem. 1982; 257:12503–12508. 6290471

[48] Tang S.N., Huang J.F. Evolution of different oligomeric glycyl-tRNA synthetases. FEBS Lett. 2005; 579:1441–1445. 15733854 10.1016/j.febslet.2005.01.045

[49] Wagar E.A., Giese M.J., Yasin B., Pang M. The glycyl-tRNA synthetase of *Chlamydia trachomatis*. J. Bacteriol. 1995; 177:5179–5185. 7665503 10.1128/jb.177.17.5179-5185.1995 PMC177304

[50] Duchene A.M., Peeters N., Dietrich A., Cosset A., Small I.D., Wintz H. Overlapping destinations for two dual targeted glycyl-tRNA synthetases in *Arabidopsis thaliana* and *Phaseolus vulgaris*. J. Biol. Chem. 2001; 276:15275–15283. 11278923 10.1074/jbc.M011525200

[51] Bullard J.M., Cai Y.C., Demeler B., Spremulli L.L. Expression and characterization of a human mitochondrial phenylalanyl-tRNA synthetase. J. Mol. Biol. 1999; 288:567–577. 10329163 10.1006/jmbi.1999.2708

[52] Schimmel P. Classes of aminoacyl-tRNA synthetases and the establishment of the genetic code. Trends Biochem. Sci. 1991; 16:1–3. 2053131 10.1016/0968-0004(91)90002-d

[53] Rodin S.N., Ohno S. Two types of aminoacyl-tRNA synthetases could be originally encoded by complementary strands of the same nucleic acid. Orig. Life Evol. Biosph. 1995; 25:565–589. 7494636 10.1007/BF01582025

[54] Carter C.W. Jr Cognition, mechanism, and evolutionary relationships in aminoacyl-tRNA synthetases. Annu. Rev. Biochem. 1993; 62:715–748. 8352600 10.1146/annurev.bi.62.070193.003435

[55] Ribas de Pouplana L., Schimmel P. Two classes of tRNA synthetases suggested by sterically compatible dockings on tRNA acceptor stem. Cell. 2001; 104:191–193. 11269237 10.1016/s0092-8674(01)00204-5

[56] Ribas de Pouplana L., Schimmel P. Aminoacyl-tRNA synthetases: potential markers of genetic code development. Trends Biochem. Sci. 2001; 26:591–596. 11590011 10.1016/s0968-0004(01)01932-6

[57] Terada T., Nureki O., Ishitani R., Ambrogelly A., Ibba M., Soll D., Yokoyama S. Functional convergence of two lysyl-tRNA synthetases with unrelated topologies. Nat. Struct. Biol. 2002; 9:257–262. 11887185 10.1038/nsb777

[58] Onesti S., Miller A.D., Brick P. The crystal structure of the lysyl-tRNA synthetase (LysU) from *Escherichia coli*. Structure. 1995; 3:163–176. 7735833 10.1016/s0969-2126(01)00147-2

[59] Carter C.W. Jr, Wills P.R. Hierarchical groove discrimination by Class I and II aminoacyl-tRNA synthetases reveals a palimpsest of the operational RNA code in the tRNA acceptor-stem bases. Nucleic. Acids. Res. 2018; 46:9667–9683. 30016476 10.1093/nar/gky600 PMC6182185

[60] Mosyak L., Reshetnikova L., Goldgur Y., Delarue M., Safro M.G. Structure of phenylalanyl-tRNA synthetase from *Thermus thermophilus*. Nat. Struct. Biol. 1995; 2:537–547. 7664121 10.1038/nsb0795-537

[61] Michalska K., Jedrzejczak R., Wower J., Chang C., Baragana B., Gilbert I.H., Forte B., Joachimiak A. *Mycobacterium tuberculosis* Phe-tRNA synthetase: structural insights into tRNA recognition and aminoacylation. Nucleic Acids Res. 2021; 49:5351–5368. 33885823 10.1093/nar/gkab272 PMC8136816

[62] Xiong Y., Steitz T.A. A story with a good ending: tRNA 3′-end maturation by CCA-adding enzymes. Curr. Opin. Struct. Biol. 2006; 16:12–17. 16364630 10.1016/j.sbi.2005.12.001

[63] Yamashita S., Tomita K. Mechanism of 3′-matured tRNA discrimination from 3′-Immature tRNA by Class-II CCA-adding enzyme. Structure. 2016; 24:918–925. 27133023 10.1016/j.str.2016.03.022

