# Supplementary material for: X-shaped structure of bacterial heterotetrameric tRNA synthetase suggests cryptic prokaryote functions and a rationale for synthetase classifications
Source: Nucleic Acids Res. 2021 Aug 14;49(17):10106–19. doi: 10.1093/nar/gkab707 (PMC8464048; doi:10.1093/nar/gkab707)
Supplement: gkab707_Supplemental_File [file gkab707_supplemental_file.pdf]

# Supplementary information

## X-shaped structure of bacterial heterotetrameric tRNA synthetase suggests cryptic prokaryote functions and a rationale for synthetase classifications

Yingchen Ju[1,2], Lu Han[1,2], Bingyi Chen[1,2], Zhiteng Luo[1,2], Qiong Gu[2], Jun Xu[2], Xiang-Lei Yang[3], Paul Schimmel[3,4,]* and Huihao Zhou[1,2,]*

[1] Guangdong Provincial Key Laboratory of Chiral Molecule and Drug Discovery, School of Pharmaceutical Sciences, Sun Yat-sen University, Guangzhou, 510006, China

[2] Research Center for Drug Discovery, School of Pharmaceutical Sciences, Sun Yat-sen University, Guangzhou, 510006, China.

[3] Department of Molecular Medicine, The Scripps Research Institute, La Jolla, CA 92037, USA.

[4] Department of Molecular Medicine, The Scripps Research Institute, Jupiter, FL 33458, USA.

* To whom correspondence should be addressed. Tel: +86 20 39943350; Email: zhuihao@mail.sysu.edu.cn

Correspondence may also be addressed to Paul Schimmel. Tel: +1 858 784 8970; Fax: +1 858 784 8990; Email: schimmel@scripps.edu

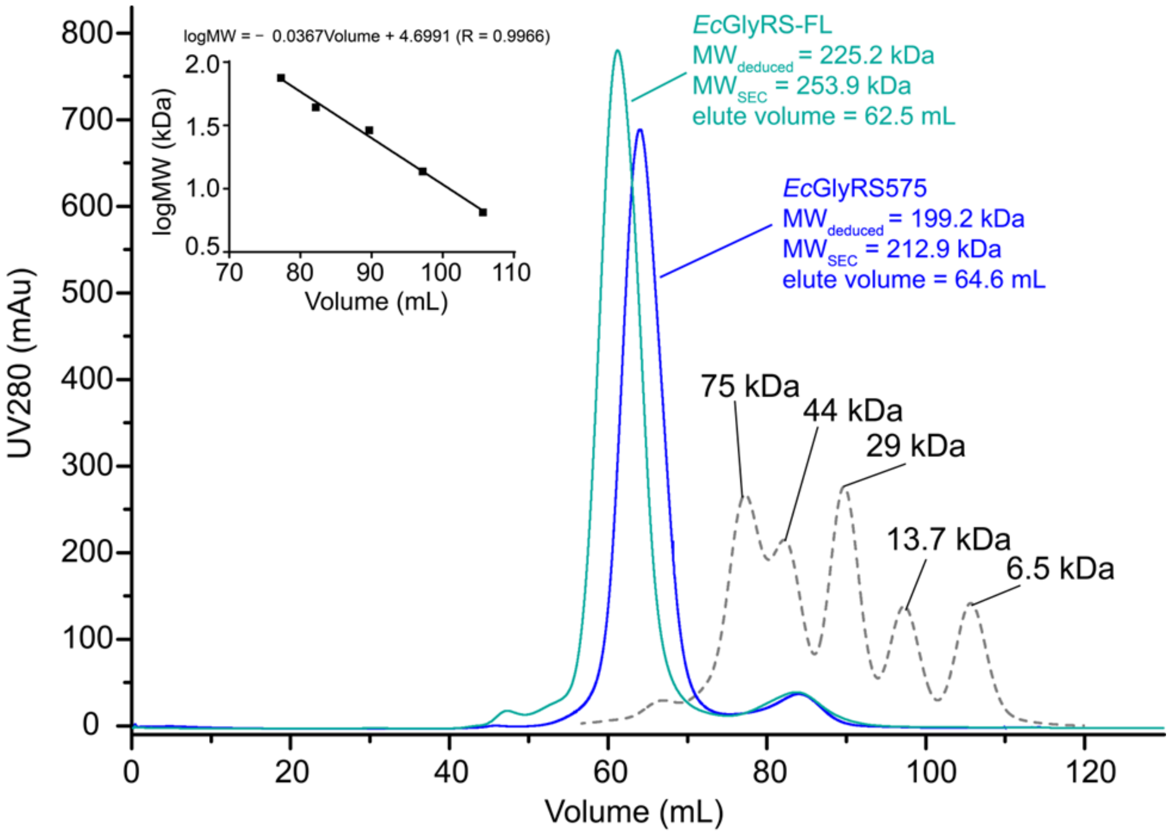


**Supplementary Figure S1. Measuring the oligomeric state of *Ec*GlyRS-FL and *Ec*GlyRS575 in solution using size-exclusion chromatography.** The apparent molecular weights of *Ec*GlyRS-FL and *Ec*GlyRS575 in solution were measured using a HiLoad 16/60 Superdex 200 pg column (Cytiva) which was calibrated with standard proteins from Gel Filtration LMW Calibration Kit (Cytiva). *Ec*GlyRS-FL was eluted as a sharp symmetric peak at 62.5 mL, which is corresponding to the molecular weight of $MW_{SEC}$ = 253.9 kDa. This number is close to the molecular weight deduced from the protein sequences of the predicted heterotetrameric form of *Ec*GlyRS-FL ($MW_{deduced}$ = 225.2 kDa), indicating that *Ec*GlyRS-FL is a heterotetramer in solution. Similarly, *Ec*GlyRS575 was eluted at 64.6 mL with a $MW_{SEC}$ = 212.9 kDa, which is close to $MW_{deduced}$ = 199.2 kDa and supports that *Ec*GlyRS575 forms a heterotetramer in solution.

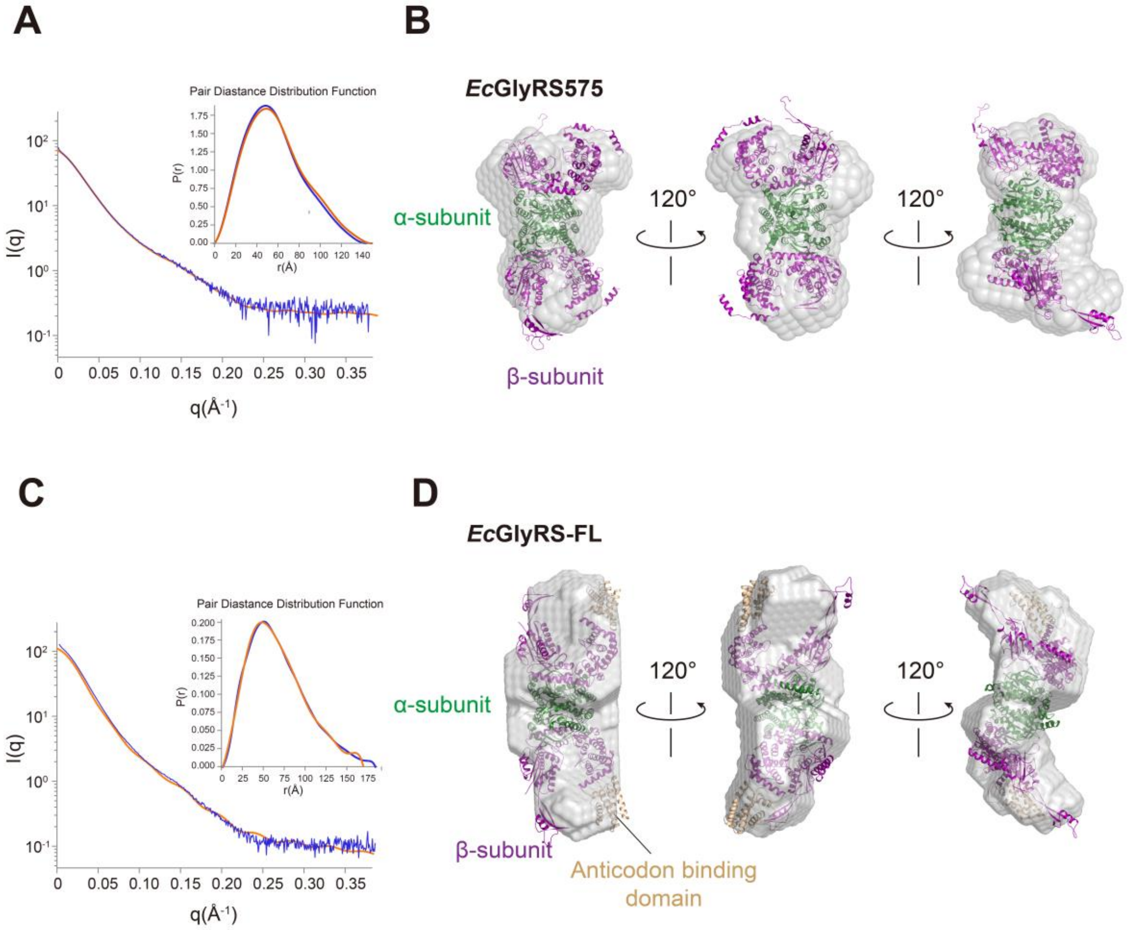


**Supplementary Figure S2. Solution Structure of *Ec*GlyRS575 and *Ec*GlyRS-FL by SAXS assays.** (**A**) The back-calculated scattering profile (orange) fits well with the experimental scattering profile (blue) for the *Ec*GlyRS575 ($\chi$=1.05, $\chi^2_{free}$=1.12). The inset shows the overlay of the pair distance distribution function (PDDF) of the crystal structure (orange) with that of SAXS experiment (blue). (**B**) The SAXS *ab initio* envelope was represented as a space-filling model (grey), and the crystal structure of *Ec*GlyRS575 fits well into this shape envelope. **(C)** The overlay of the scattering profiles of the modeled structure (orange) and SAXS experiment (blue) for *Ec*GlyRS-FL ($\chi$=1.36, $\chi^2_{free}$=1.86). The inset shows the overlay of the modeled structure (orange) with experimental (blue) PDDFs. (**D**) The structure of *Ec*GlyRS-FL with the modeled ABD (wheat) fits well in the SAXS *ab initio* envelope.

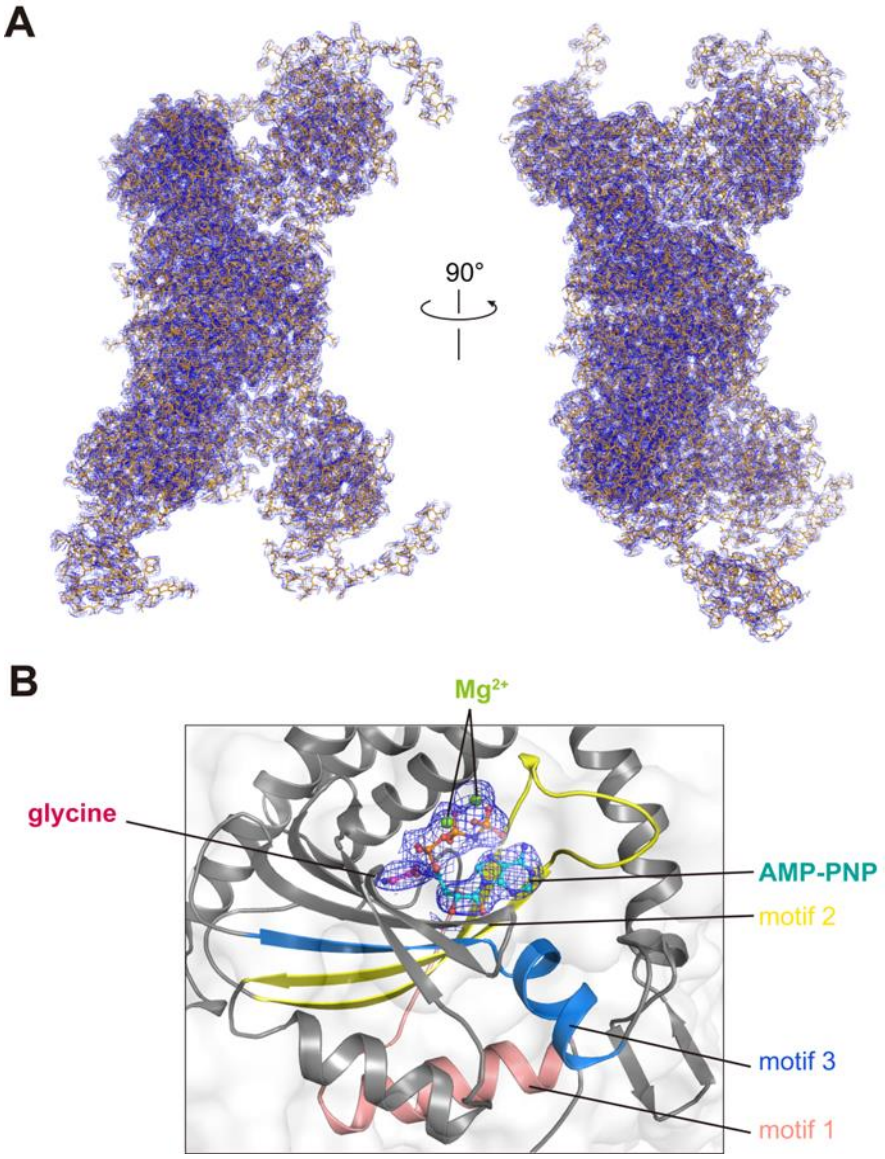


**Supplementary Figure S3. Electron density map of *Ec*GlyRS575 structure.** (**A**) The $2F_o$-$F_c$ electron density map of the protein chain. (**B**) The $2F_o$-$F_c$ electron density maps for AMP-PNP, glycine, and $Mg^{2+}$ are shown as blue meshes and contoured at 1.0 σ in the aminoacylation pocket of *Ec*GlyRS575. Three signature motifs of class II aaRSs on the α-subunit are labeled (motif 1, res. 9-32; motif 2, res. 54-88; motif 3, res. 160-175).

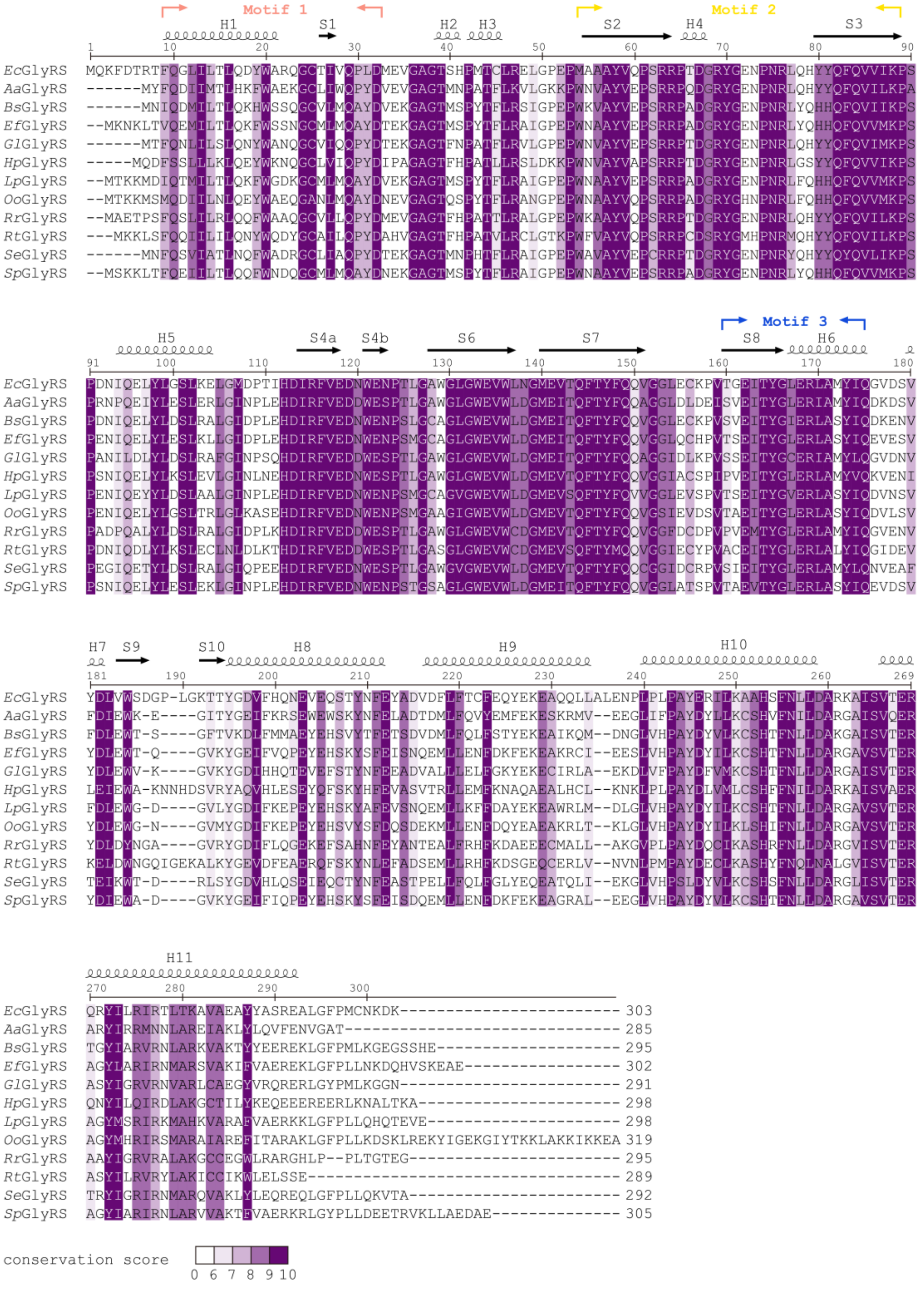


**Supplementary Figure S4. Sequence alignments of α-subunits from *Ec*GlyRS and homologs.** Protein sequences of *E. coli* GlyRS (*Ec*GlyRS, UniProtKB ID: P00960), *Aquifex aeolicus* GlyRS (*Aa*GlyRS, UniProtKB ID: O67081), *Bacillus subtilis* GlyRS (*Bs*GlyRS, UniProtKB ID: P54380), *Enterococcus faecalis* GlyRS (*Ef*GlyRS, UniProtKB ID: Q831U2), *Geobacter lovleyi* GlyRS (*Gl*GlyRS, UniProtKB ID: B3E622), *Helicobacter pylori* GlyRS (*Hp*GlyRS, UniProtKB ID:

B5Z7W3), *Lactobacillus paracasei* GlyRS (*Lp*GlyRS, UniProtKB ID: Q038U2), *Oenococcus oeni* GlyRS (*Oo*GlyRS, UniProtKB ID: Q04F71), *Rhodospirillum rubrum* GlyRS (*Rr*GlyRS, UniProtKB ID: Q2RQ44), *Rickettsia typhi* GlyRS (*Rt*GlyRS, UniProtKB ID: Q68VR3), *Synechococcus elongatus* GlyRS (*Se*GlyRS, UniProtKB ID: Q31KD2) and *Streptococcus pneumoniae* GlyRS (*Sp*GlyRS, UniProtKB ID: B8ZL21) were aligned using Clustal Omega program(1). The residue numbering and secondary structures corresponding to *Ec*GlyRS are displayed above the sequences. The conservation scores were calculated by the program Jalview(2) and exhibited in various shades of purples. The signature motifs 1, 2, and 3 conserved in class II aaRSs are marked with salmon, yellow and blue arrows, respectively.

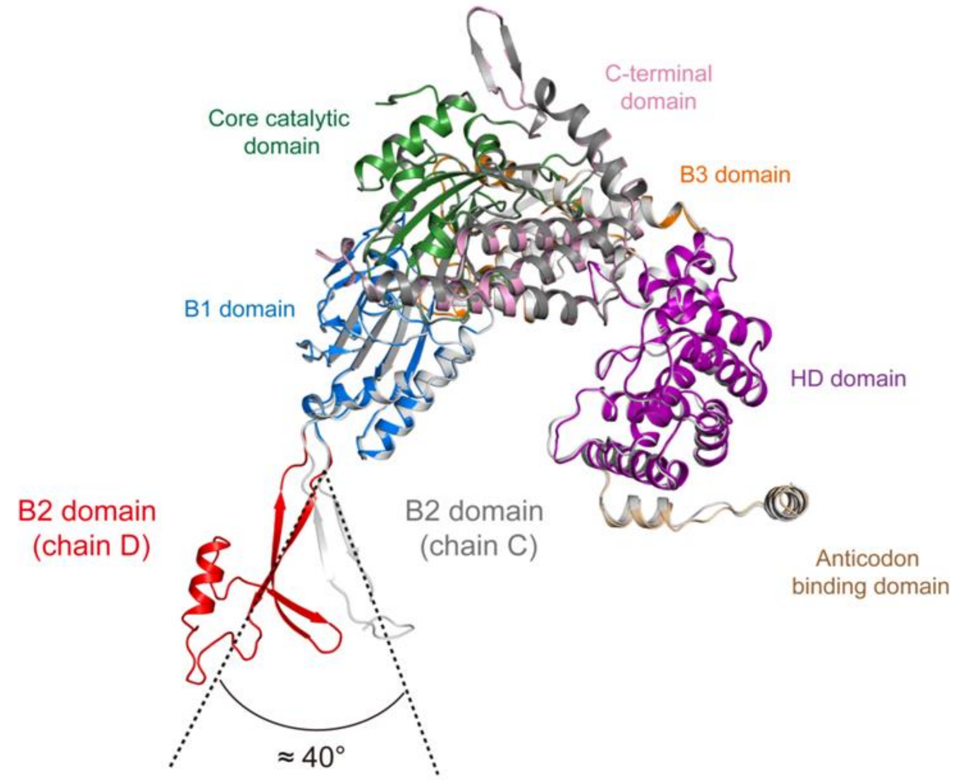


**Supplementary Figure S5. The structural flexibility of the B2 domain.** The structure superposition of the two protomers (colored the same as in Figure 1B) reveals that both protomers are almost identical except that their B2 domains undergo large conformation movement between two protomers.

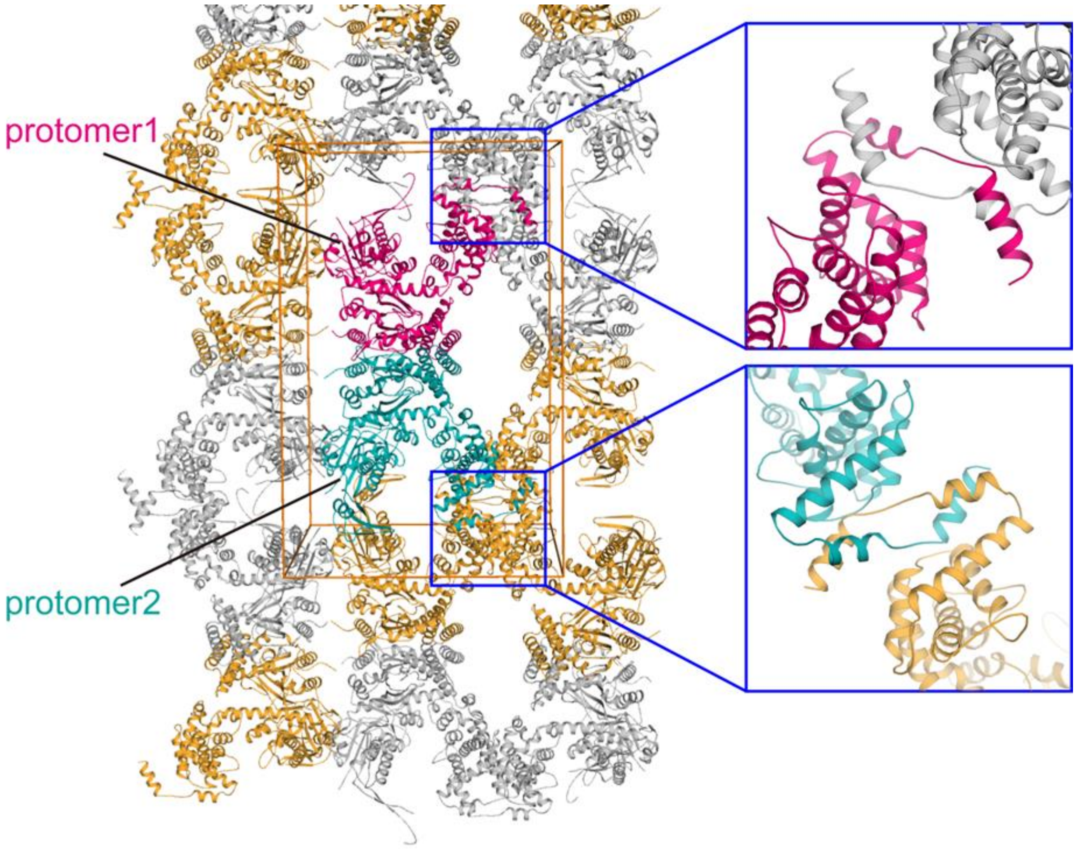


**Supplementary Figure. S6. Domain-swapping interactions between the residual ABD sequences of two *Ec*GlyRS575 proteins from adjacent asymmetric units contribute to crystal packing.** The yellow cube represents an asymmetric unit that contains one *Ec*GlyRS575 molecule consisting of two protomers (colored in magenta for protomer1 and cyan for protomer 2). The two protomers of the adjacent *Ec*GlyRS575 molecules are colored in gray and gold, respectively. The zoom-in views show the formation of domain-swapping interactions between adjacent *Ec*GlyRS575 molecules.

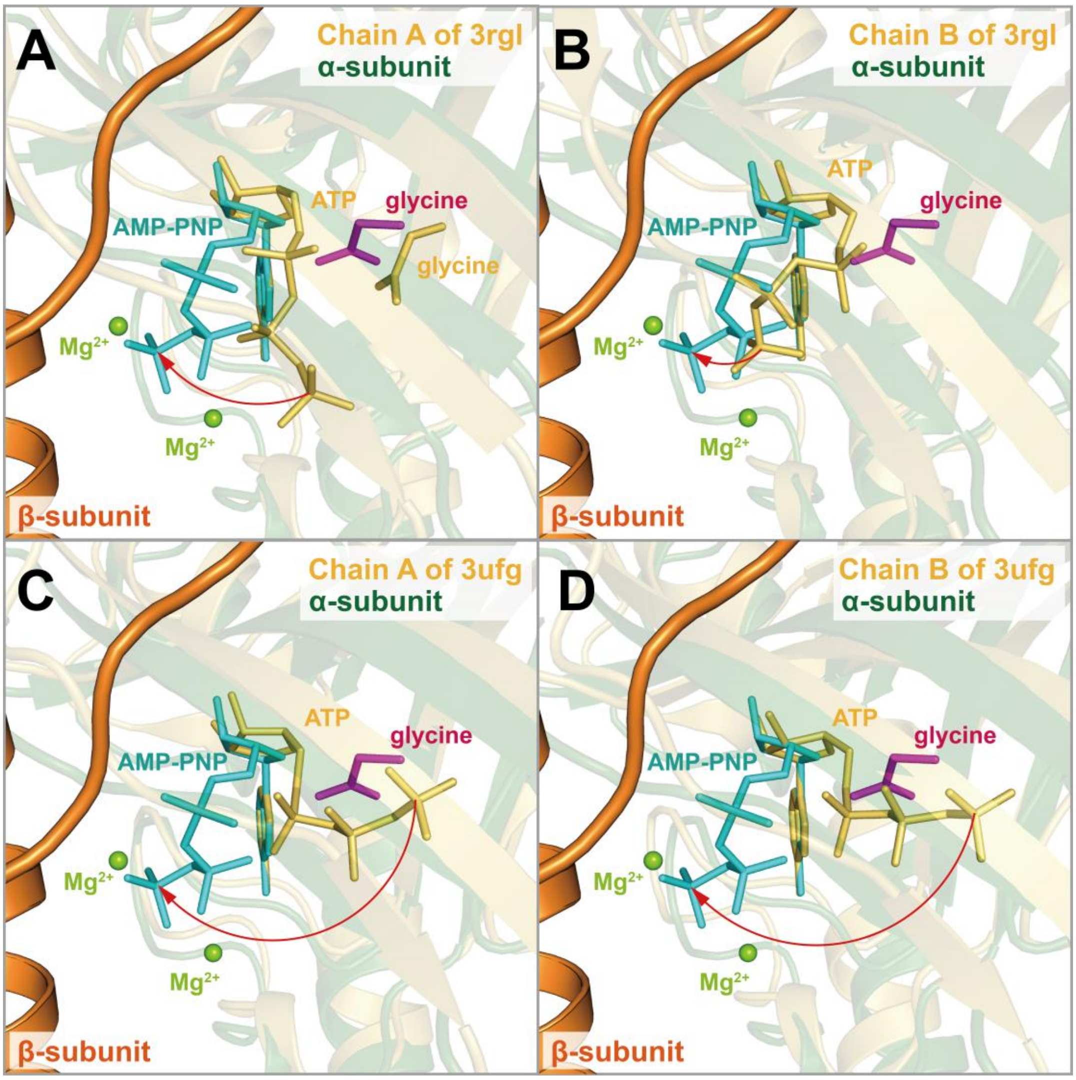


**Supplementary Figure S7. The phosphate groups of AMP-PNP rotate toward the β-subunit.** Structural comparison of the aminoacylation pockets between *Ec*GlyRS and *Cj*GlyRS-ATP-glycine and *Cj*GlyRS-ATP complexes (PDB: 3grl and 3ufg, colored yellow), and the major conformation differences of the phosphate groups are indicated by red arrows.

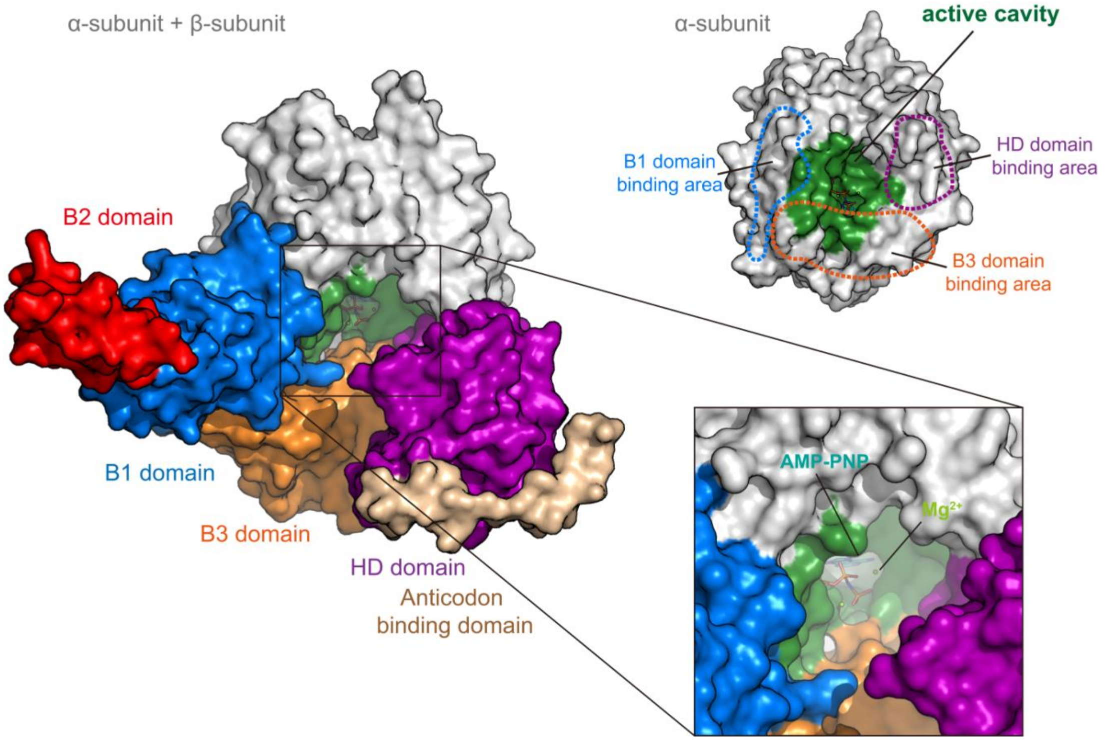


**Supplementary Figure S8. The binding of B1, B3 and HD domains of β-subunit to the areas surrounding of the active cavity on the α-subunit results in the formation of a deeper and better-covered aminoacylation pocket.**

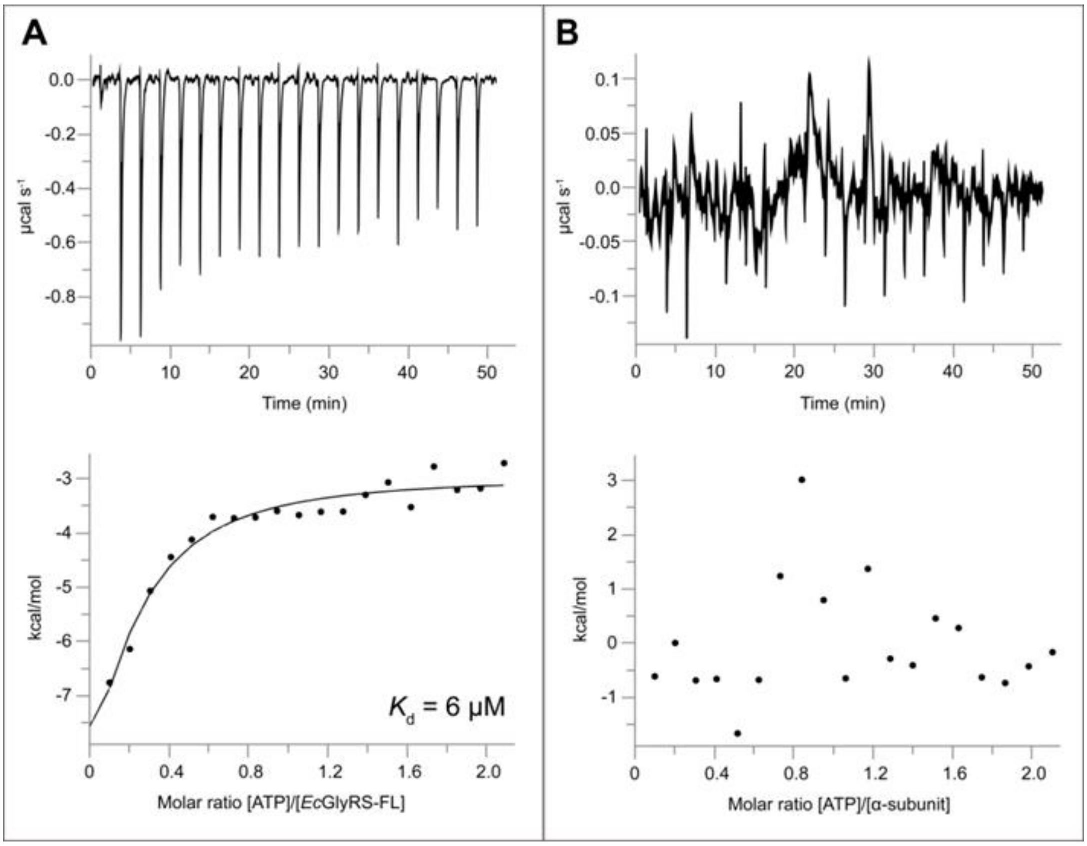


**Supplementary Figure S9. ITC titrations of ATP to *Ec*GlyRS-FL or α-subunit.** (**A**) ITC titration of ATP into *Ec*GlyRS-FL. The top panel shows raw thermogram and bottom panel shows the binding isotherm fitted to a single-site model (**B**) Titration of ATP into purified α-subunit of *Ec*GlyRS. Bottom panel isotherm was very close to baseline, indicating that the binding affinity between ATP and α-subunit alone is very low.

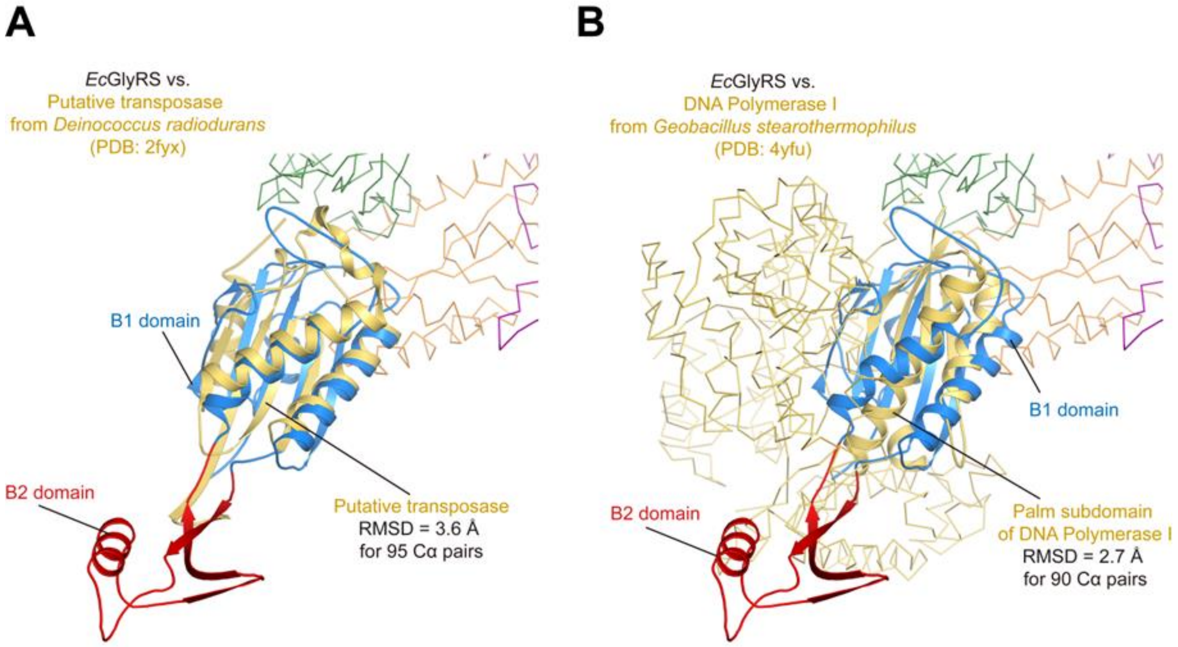


**Supplementary Figure S10. Structural superposition of the B1-B2 domain and its homologs.** (**A**) The structural superposition of the B1-B2 domains of the β-subunit (colored the same as Fig. 1A) and a putative transposase from *Deinococcus radiodurans* (PDB: 2fyx, golden). (**B**) The B1-B2 domain is superimposed to the DNA-grasping palm subdomain of the DNA polymerase I from *Geobacillus stearothermophilus*. The domains used in structural superposition are shown as cartoons, and the rest parts of the structures are presented as ribbons.

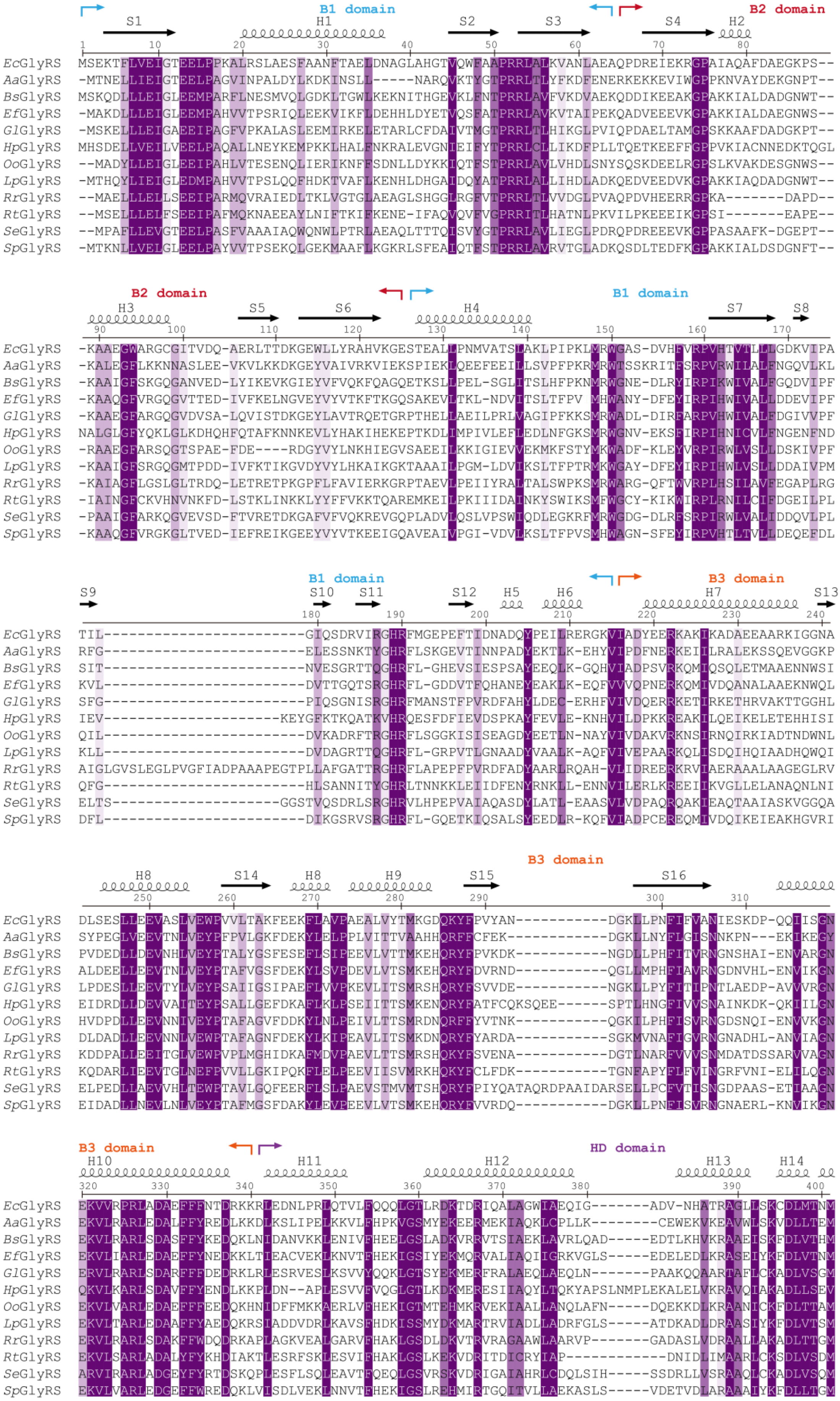
B1 domain
B2 domain
B3 domain
HD domain
EcGlyRS
AaGlyRS
BsGlyRS
EfGlyRS
GlGlyRS
HpGlyRS
OoGlyRS
LpGlyRS
RrGlyRS
RtGlyRS
SeGlyRS
SpGlyRS

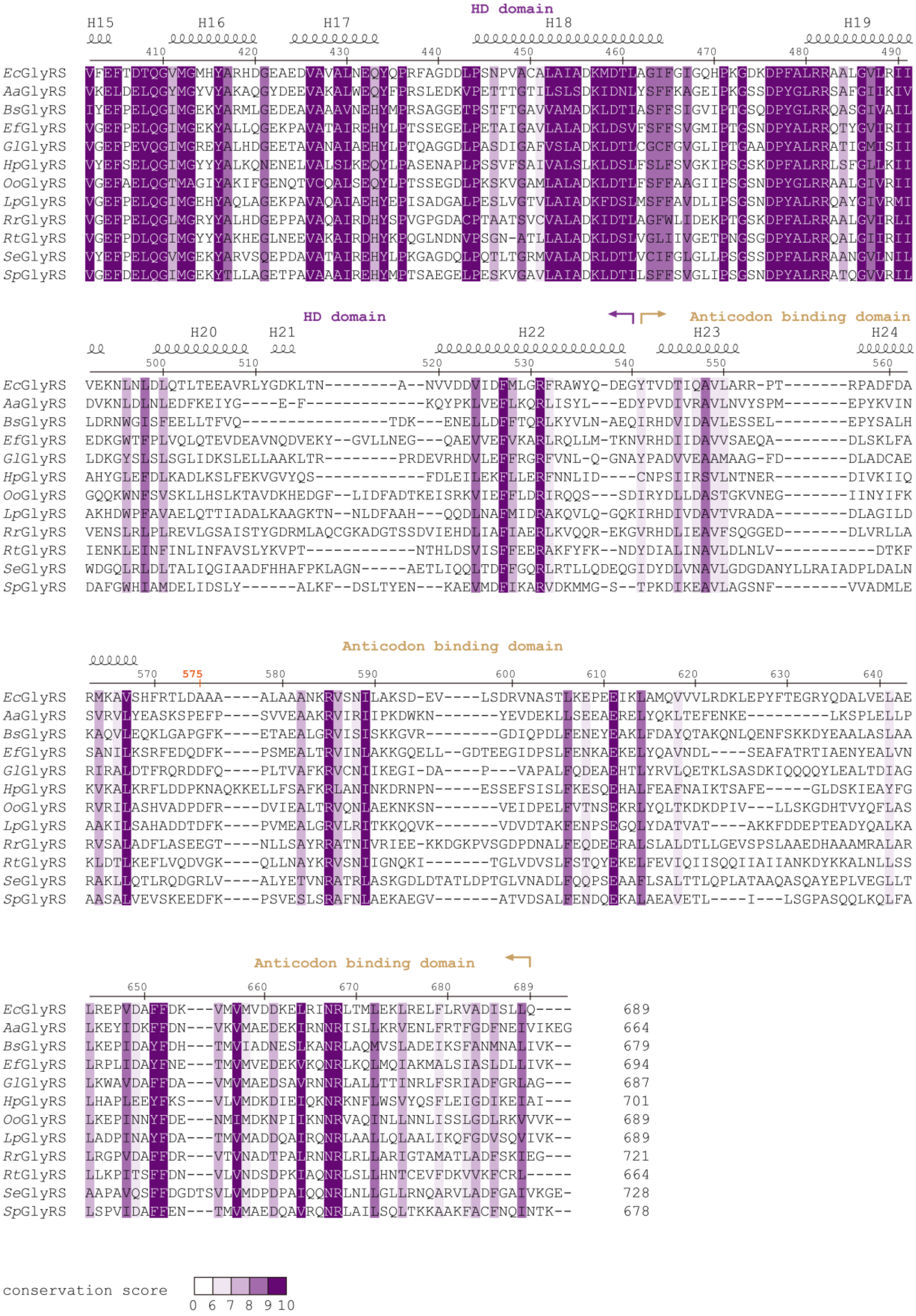
HD domain
H15 H16 H17 H18 H19
410 420 430 440 450 460 470 480 490
EcGlyRS VFEFTDTQGVMGMHYARHDGEAEDVAVALNEQYQPRFAGDDLPSNPVACALAIADKMDTIAGIFGIGQHPKGDKDPFALRRAALGVLRII
AaGlyRS VKELDELQGYMGYVYAKAQGYDEEVAKALWEQYFPRSLEDKVPETTTGTILSLSDKIDNIYSFFKAGEIPKGSSDPYGLRRSAFGIIKIV
BsGlyRS IYEFPELQGIMGEKYARMLGEDEAVAAAVNEHYMPRSAGGETPSTFTGAVVAMADKLDTIASFFSIGVIPTGSQDPYGLRRQASGIVAIL
EfGlyRS VGEFPELQGIMGEKYALLQGEKPAVATAIREHYLPTSSEGELPETAIGAVLALADKLDSVFSFFSVGMIPTGSNDPYALRRQTYGVIRII
GlGlyRS VGEFPEVQGIMGREYALHDGEETAVANAIAEHYLPTQAGGDLPASDIGAFVSLADKLDTLCGCFGVGLIPTGAADPYALRRATIGMISII
HpGlyRS VYEFSELQGIMGYYYALKQNENELVALSLKEQYLPASENAPLPSSVFSAIVALSLKLDSIFSLFSVGKIPSGSKDPFALRRLSFGLLKII
OoGlyRS VGEFAELQGTMAGIYAKIFGENQTVCQALSEQYLPTSSEGDLPKSKVGAMLALADKLDTLFSFFAAGIIPSGSNDPYGLRRAALGIVRII
LpGlyRS VGEFPELQGIMGEHYAQLAGEKPAVAQAIAEHYEPISADGALPESLVGTVLAIADKFDSIMSFFAVDLIPSGSNDPYALRRQAYGIVRMI
RrGlyRS VGEFPELQGLMGRYYALHDGEPPAVAQAIRDHYSPVGPGDACPTAATSVCVALADKIDTLAGFWLIDEKPTGSKDPFALRRAALGVIRLI
RtGlyRS VGEFPDLQGIMGYYYAKHEGLNEEVAKAIRDHYKPQGLNDNVPSGN-ATLLALADKLDSIVGLIIVGETPNGSGDPYALRRQALGIIRII
SeGlyRS VYEFPELQGVMGEKYARVSQEPDAVATAIREHYLPKGAGDQLPQTLTGRMVALADRLDTLVCIFGLGLLPSGSSDPFALRRAANGVLNIL
SpGlyRS VGEFDELQGIMGEKYTLLAGETPAVAAAIREHYMPTSAEGELPESKVGAVLAIADKLDTILSFFSVGLIPSGSNDPYALRRATQGVVRIL
HD domain
Anticodon binding domain
H20 H21 H22 H23 H24
500 510 520 530 540 550 560
EcGlyRS VEKNLNLDLQTLTEEAVRLYGDKLTN--------A--NVVDDVIDFMLGRFRAWYQ-DEGYTVDTIQAVLARR-PT-------RPADFDA
AaGlyRS DVKNLDLNLEDFKEIYG----E-F-------------KQYPKLVEFLKQRLISYL---EDYPVDIVRAVLNVYSPM-------EPYKVIN
BsGlyRS LDRNWGISFEELLTFVQ----------------TDK---ENELLDFFTQRLKYVLN-AEQIRHDVIDAVLESSEL--------EPYSALH
EfGlyRS EDKGWTFPLVQLQTEVDEAVNQDVEKY--GVLLNEG---QAEVVEFVKARLRQLLM-TKNVRHDIIDAVVSAEQA--------DLSKLFA
GlGlyRS LDKGYSLSLSGLIDKSLELLAAKLTR--------PRDEVRHDVLEFFRGRFVNL-Q-GNAYPADVVEAAMAAG-FD-------DLADCAE
HpGlyRS AHYGLEFDLKADLKSLFEKVGVYQS------------FDLEILEKFLLERFNNLID----CNPSIIRSVLNTNER--------DIVKIIQ
OoGlyRS GQQKWNFSVSKLLHSLKTAVDKHEDGF--LIDFADTKEISRKVIEFFLDRIRQQS---SDIRYDLLDASTGKVNEG-------IINYIFK
LpGlyRS AKHDWPFAVAELQTTIADALKAAGKTN--NLDFAAH---QQDLNAFMIDRAKQVLQ-GQKIRHDIVDAVTVRADA--------DLAGILD
RrGlyRS VENSLRLPLREVLGSAISTYGDRMLAQCGKADGTSSDVIEHDLIAFIAERLKVQQR-EKGVRHDLIEAVFSQGGED-------DLVRLLA
RtGlyRS IENKLEINFINLINFAVSLYKVPT-------------NTHLDSVISFFEERAKFYFK--NDYDIALINAVLDLNLV-----------DTKF
SeGlyRS WDGQLRLDLTALIQGIAADFHHAFPKLAGN-----AETLIQQLTDFFGQRLRTLLQDEQGIDYDLVNAVLGDGDANYLLRAIADPLDALN
SpGlyRS DAFGWHIAMDELIDSLY------ALKF--DSLTYEN---KAEVMDEIKARVDKMMG-S--TPKDIKEAVLAGSNF--------VVADMLE
Anticodon binding domain
570 575 580 590 600 610 620 630 640
EcGlyRS RMKAVSHFRTLDAAA----ALAAANKRVSNILAKSD-EV----LSDRVNASTLKEPEEIKLAMQVVVLRDKLEPYFTEGRYQDALVELAE
AaGlyRS SVRVLYEASKSPEFP----SVVEAAKRVIRIIPKDWKN-------YEVDEKLLSEEAEREIYQKLTEFENKE---------LKSPLELLP
BsGlyRS KAQVLEQKLGAPGFK----ETAEALGRVISISKKGVR--------GDIQPDLFENEYEAKLFDAYQTAKQNLQENFSKKDYEAALASLAA
EfGlyRS SANILKSRFEDQDFK----PSMEALTRVINIAKKGQELL--GDTEEGIDPSLFENKAEKELYQAVNDL----SEAFATRTIAENYEALVN
GlGlyRS RIRALDTFRQRDDFQ----PLTVAFKRVCNIIKEGI-DA----P---VAPALFQDEAEHTLYRVLQETKLSASDKIQQQQYLEALTDIAG
HpGlyRS KVKALKRFLDDPKNAQKKELLFSAFKRLANINKDRNPN-----ESSEFSISLFKESQEHALFEAFNAIKTSAFE-----GLDSKIEAYFG
OoGlyRS RVRILASHVADPDFR----DVIEALTRVQNIAEKNKSN-------VEIDPELFVTNSEKRLYQLTKDKDPIV---LLSKGDHTVYQFLAS
LpGlyRS AAKILSAHADDTDFK----PVMEALGRVLRITKKQQVK-------VDVDTAKFENPSEGQLYDATVAT----AKKFDDEPTEADYQALKA
RrGlyRS RVSALADFLASEEGT----NLLSAYRRATNIVRIEE-KKDGKPVSGDPDNALFEQDEERALSLALDTLLGEVSPSLAAEDHAAAMRALAR
RtGlyRS KLDTLKEFLVQDVGK----QLLNAYKRVSNIIGNQKI-------TGLVDVSLFSTQYEKELFEVIQIISQQIIAIIANKDYKKALNLLSS
SeGlyRS RAKLLQTLRQDGRLV----ALYETVNRATRIASKGDLDTATLDPTGLVNADLFQQPSEAAFLSALTTLQPLATAAQASQAYEPLVEGLLT
SpGlyRS AASALVEVSKEEDFK----PSVESLSRAFNLAEKAEGV-------ATVDSALFENDQEKALAEAVETL----I---LSGPASQQLKQLFA
Anticodon binding domain
650 660 670 680 689
EcGlyRS LREPVDAFFDK---VMVMVDDKELRINRLTMLEKLRELFLRVADISLLQ---- 689
AaGlyRS LKEYIDKFFDN---VKVMAEDEKIRNNRISLLKRVENLFRTFGDFNEIVIKEG 664
BsGlyRS LKEPIDAYFDH---TMVIADNESLKANRLAQMVSLADEIKSFANMNALIVK-- 679
EfGlyRS LRPLIDAYFNE---TMVMVEDEKVKQNRLKQLMQIAKMALSIASLDLLIVK-- 694
GlGlyRS LKWAVDAFFDA---VMVMAEDSAVRNNRLALLTTINRLFSRIADFGRLAG--- 687
HpGlyRS LHAPLEEYFKS---VLVMDKDIEIQKNRKNFLWSVYQSFLEIGDIKEIAI--- 701
OoGlyRS LKEPINNYFDE---NMIMDKNPIIKNNRVAQINLLNNLISSLGDLRKVVVK-- 689
LpGlyRS LADPINAYFDA---TMVMADDQAIRQNRLAALLQLAALIKQFGDVSQVIVK-- 689
RrGlyRS LRGPVDAFFDR---VTVNADTPALRNNRLRLLARIGTAMATLADFSKIEG--- 721
RtGlyRS LLKPITSFFDN---VLVNDSDPKIAQNRLSLLHNTCEVFDKVVKFCRL----- 664
SeGlyRS AAPAVQSFFDGDTSVLVMDPDPAIQQNRLNLLGLLRNQARVLADFGAIVKGE- 728
SpGlyRS LSPVIDAFFEN---TMVMAEDQAVRQNRLAILSQLTKKAAKFACFNQINTK-- 678
conservation score
0 6 7 8 9 10

**Supplementary Figure S11. Sequence alignments of β-subunit from *Ec*GlyRS and homologs.** Protein sequences of the β-subunits of *Ec*GlyRS (UniProtKB ID: P00961), *Aa*GlyRS (UniProtKB ID: O67898), *Bs*GlyRS (UniProtKB ID: P54381), *Ef*GlyRS (UniProtKB ID: Q831U3), *Gl*GlyRS, (UniProtKB ID:B3E621), *Hp*GlyRS, (UniProtKB ID: B5Z7X4), *Oo*GlyRS, (UniProtKB ID: Q04F69), *Lp*GlyRS (UniProtKB ID: IDQ038U3), *Rr*GlyRS (UniProtKB ID: Q2RQ43), *Rt*GlyRS (UniProtKB ID: Q68VR4), *Se*GlyRS (UniProtKB ID: Q31SB9) and *Sp*GlyRS (UniProtKB ID: B8ZL20) were aligned using Clustal Omega program(1). The residue numbering and secondary structures corresponding to *Ec*GlyRS are displayed above the sequence. The conservation scores were calculated by the program Jalview(2) and exhibited in various shades of purples.

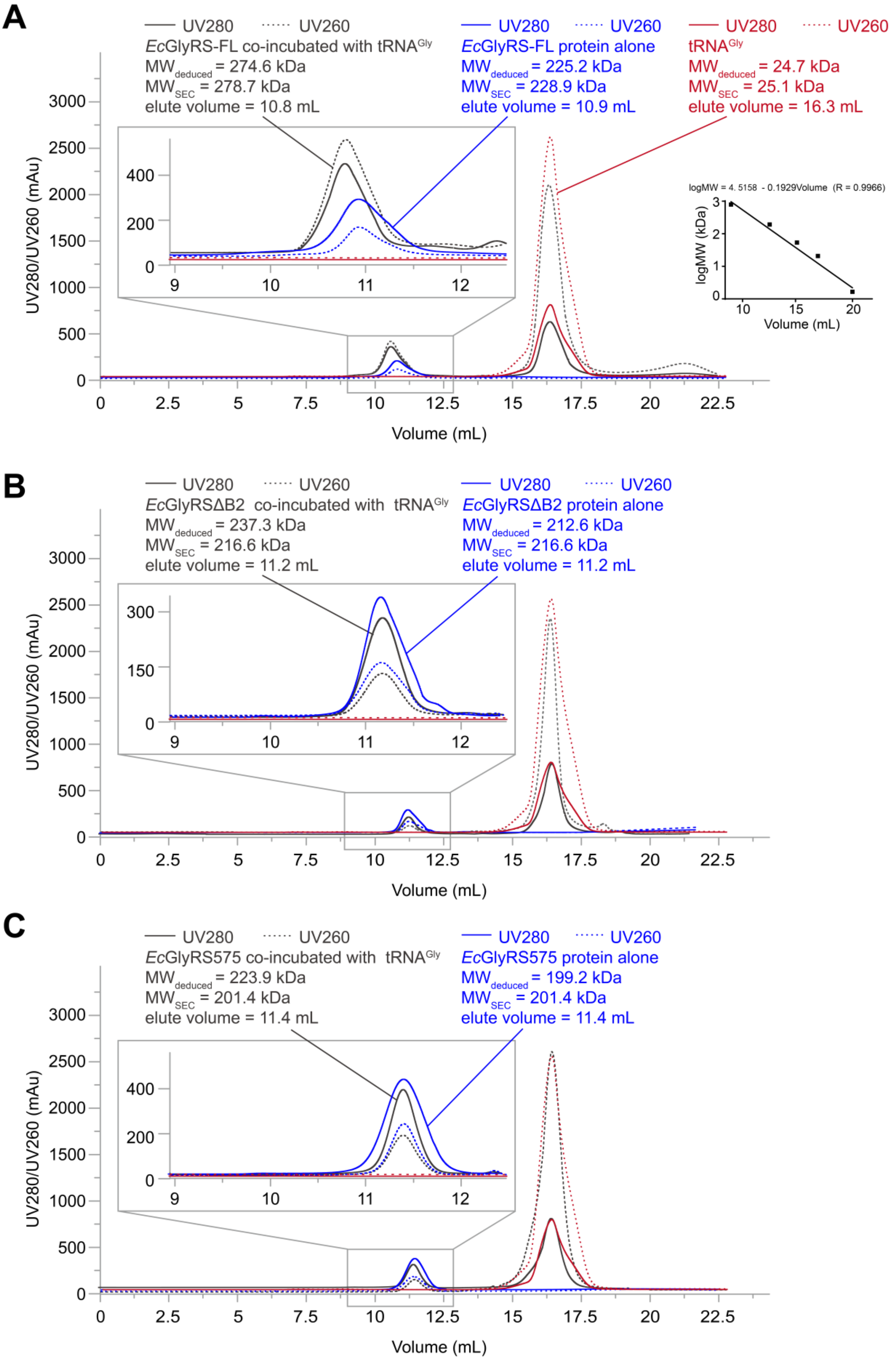

A
UV280 UV260
EcGlyRS-FL co-incubated with tRNAGly
MWdeduced = 274.6 kDa
MWSEC = 278.7 kDa
elute volume = 10.8 mL
UV280 UV260
EcGlyRS-FL protein alone
MWdeduced = 225.2 kDa
MWSEC = 228.9 kDa
elute volume = 10.9 mL
UV280 UV260
tRNAGly
MWdeduced = 24.7 kDa
MWSEC = 25.1 kDa
elute volume = 16.3 mL
logMW = 4. 5158 - 0.1929Volume (R = 0.9966)
logMW (kDa)
Volume (mL)
UV280/UV260 (mAu)
Volume (mL)
B
UV280 UV260
EcGlyRSΔB2 co-incubated with tRNAGly
MWdeduced = 237.3 kDa
MWSEC = 216.6 kDa
elute volume = 11.2 mL
UV280 UV260
EcGlyRSΔB2 protein alone
MWdeduced = 212.6 kDa
MWSEC = 216.6 kDa
elute volume = 11.2 mL
UV280/UV260 (mAu)
Volume (mL)
C
UV280 UV260
EcGlyRS575 co-incubated with tRNAGly
MWdeduced = 223.9 kDa
MWSEC = 201.4 kDa
elute volume = 11.4 mL
UV280 UV260
EcGlyRS575 protein alone
MWdeduced = 199.2 kDa
MWSEC = 201.4 kDa
elute volume = 11.4 mL
UV280/UV260 (mAu)
Volume (mL)

**Supplementary Figure S12. Binding of full length and truncated *Ec*GlyRS to $tRNA^{Gly}$ is analyzed by using size-exclusion chromatography.** The binding assays were performed by using a Superdex 200 increase 10/30 column (Cytiva), which was calibrated with standard proteins from Gel Filtration LMW Calibration Kit (Cytiva). Full and dash lines represent the absorption at a wavelength of 280 and 260 nm, respectively. The molar ratio for the *Ec*GlyRS protein and $tRNA^{Gly}$ is 4:1. As shown in (**A**), after 30 min co-incubation at room temperature, *Ec*GlyRS-FL and $tRNA^{Gly}$ were able to form a peak (gray) which shifts to the left compared to the peak of *Ec*GlyRS-FL alone (blue). And the UV260 of the gray peak increased significantly compared to the blue peak, indicating the complex formation of *Ec*GlyRS-FL and $tRNA^{Gly}$. After using the same co-incubation process as *Ec*GlyRS-FL, in contrast, the *Ec*GlyRSΔB2 (**B**) and *Ec*GlyRS575 (**C**) protein could not form a complex with the same amount of $tRNA^{Gly}$, as their gray peaks in gel-filtration showed almost the same location and intensity with the blue peak. These results showed the importance of the B2 domain and ABD of *Ec*GlyRS in tRNA binding.

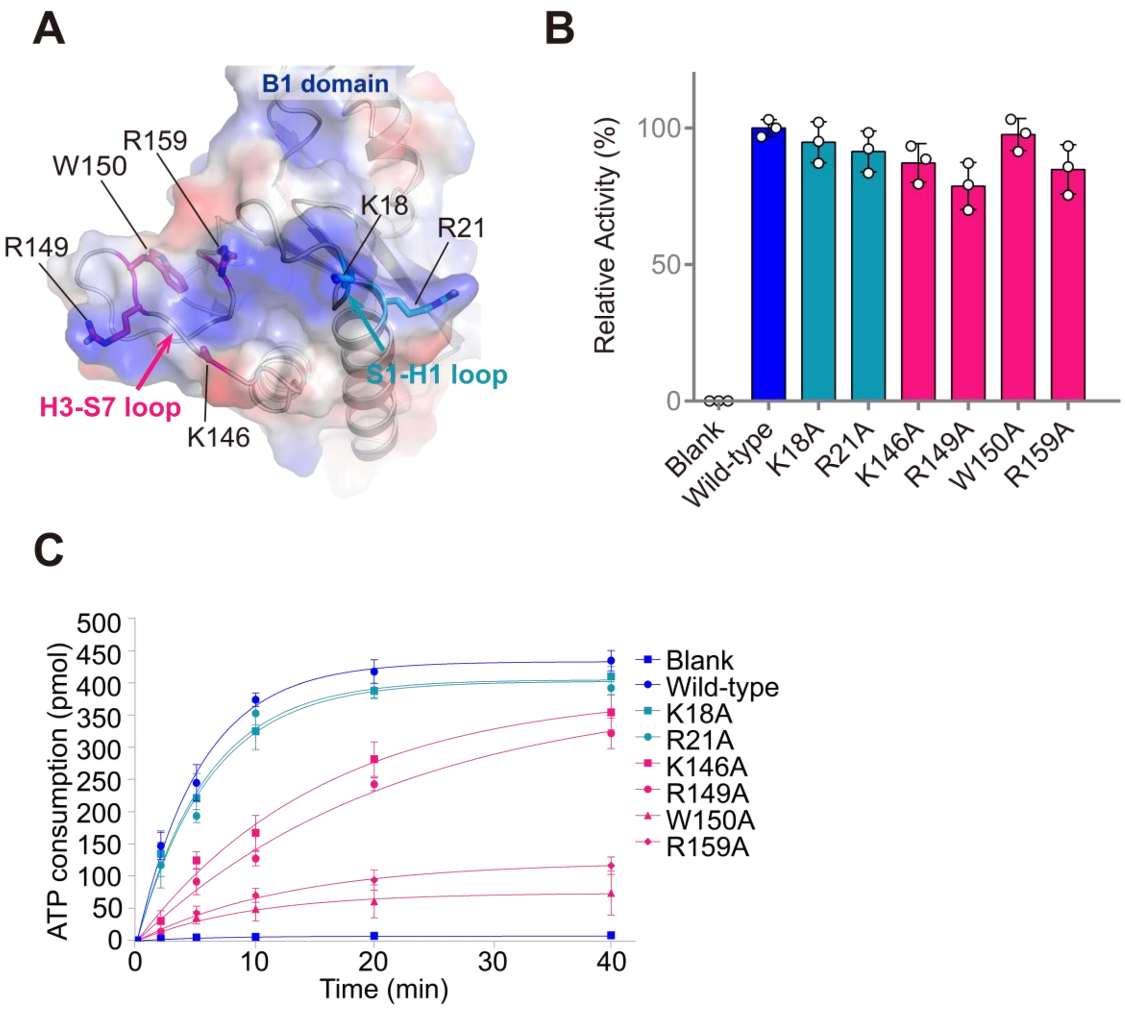


**Supplementary Figure S13. The structural and mutagenesis analysis of the B1 domain surface facing tRNA acceptor stem.** (**A**) The electrostatic surface of the B1 domain facing tRNA acceptor stem. The positively charged residues in the S1-H1 loop and H3-S7 are shown as sticks. (**B**) The glycine activation of wild-type *Ec*GlyRS and *Ec*GlyRS variants with single mutations on the B1 domain were measured by a continuous spectrophotometric assay. The activity of the wild-type enzyme is normalized as 100%. The experiments were repeated three times, and the blank circles indicate the results for each experiment. The error bars are SD. (**C**) The aminoacylation activities of the wild-type *Ec*GlyRS and its variants with single mutations in the B1 domain.

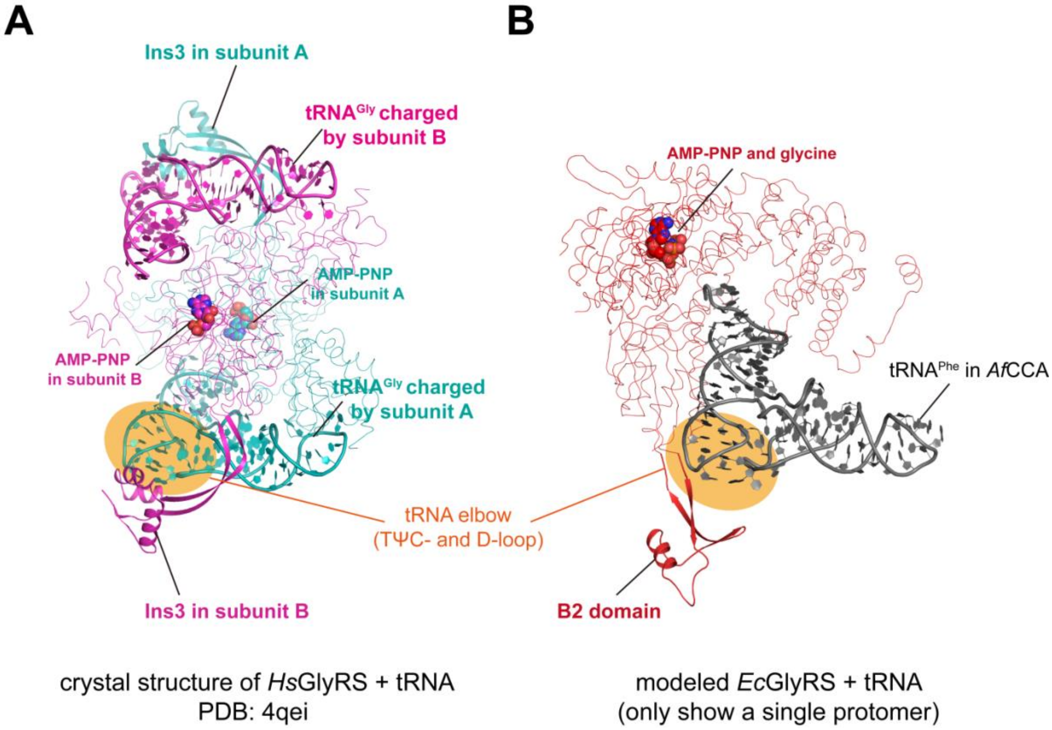


**Supplementary Figure S14. Structural comparison between the Ins3 domain of *Hs*GlyRS and the B2 domain of *Ec*GlyRS in tRNA$^{Gly}$ binding.** (**A**) The structure of *Hs*GlyRS in complex with tRNA$^{Gly}$ (PDB: 4qei) reveals that the Ins3 domain (shown as cartoon) could bind to the elbow region of tRNA$^{Gly}$. Notably, a cross-subunit tRNA$^{Gly}$-binding mode was employed by *Hs*GlyRS, that the acceptor arm and anticodon of a tRNA$^{Gly}$ molecule are recognized by one subunit of *Hs*GlyRS homodimer while its elbow region is recognized by Ins3 from the other subunit. (**B**) The B2 domain (red) of *Ec*GlyRS575 is close to the elbow region of the substrate tRNA$^{Gly}$ according to structural modeling based on the crystal structure of *Af*CCA-tRNA$^{Phe}$ complex (PDB: 1sz1), and with its conformation dynamics, B2 is likely able to contact the elbow region of tRNA$^{Gly}$. Different to *Hs*GlyRS-tRNA$^{Gly}$ complex, the tRNA$^{Gly}$ molecule will be recognized by different domains from a single protomer of *Ec*GlyRS575.

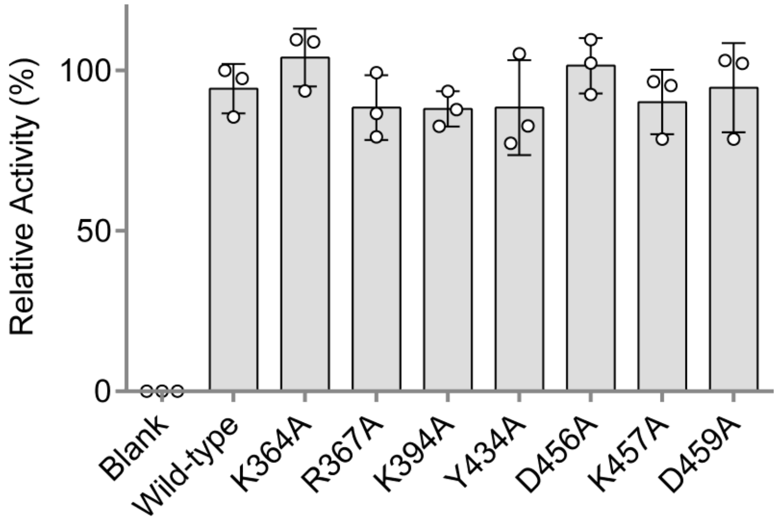


**Supplementary Figure S15. The glycine activation activity of *Ec*GlyRS-FL and its HD domain variants.** The amino acid activation activity of wild-type *Ec*GlyRS-FL and its variants in the HD domain were measured by a continuous spectrophotometric assay, and the results confirmed that the single site-directed mutations of the cavity-forming residues in the HD domain does not affect the first step of the catalysis.

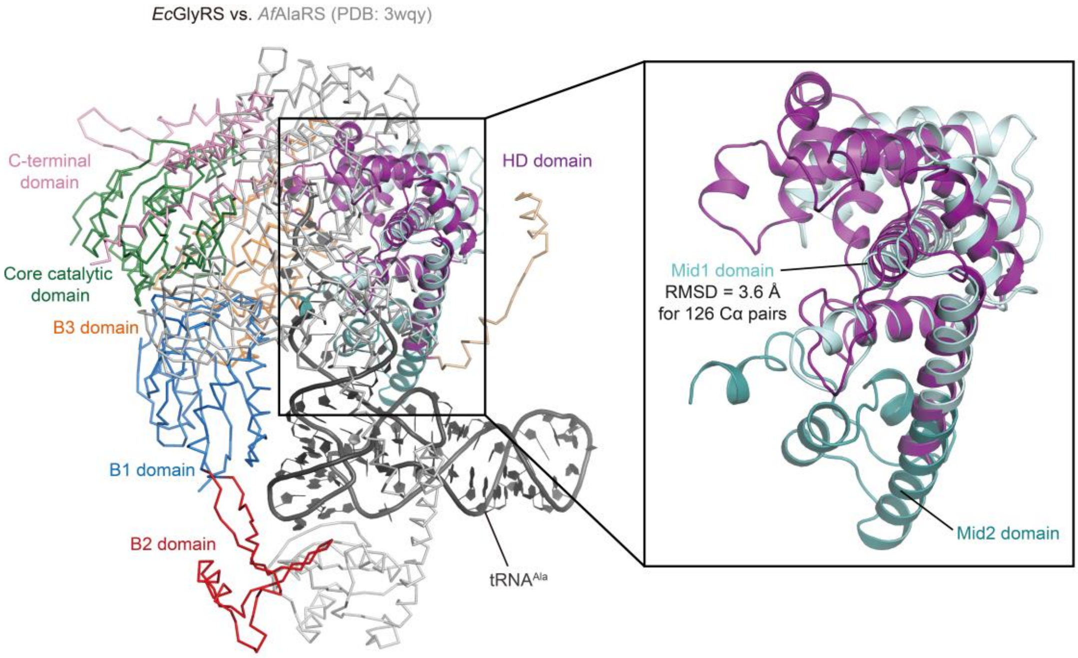


**Supplementary Figure S16.The structural superposition between the HD domain and the Mid1 subdomain of *Archaeoglobus fulgidus* AlaRS (*Af*AlaRS) in complex with tRNA$^{Ala}$ (PDB: 3wqy).** The Mid 1 (palecyan) in the tRNA-recognition domain of the *Af*AlaRS and tRNA$^{Ala}$ (black) are shown as the cartoon. The HD domain (purple) is shown as cartoon, and other parts of the *Ec*GlyRS (a protomer only) are shown as ribbons and colored the same as Figure1B.

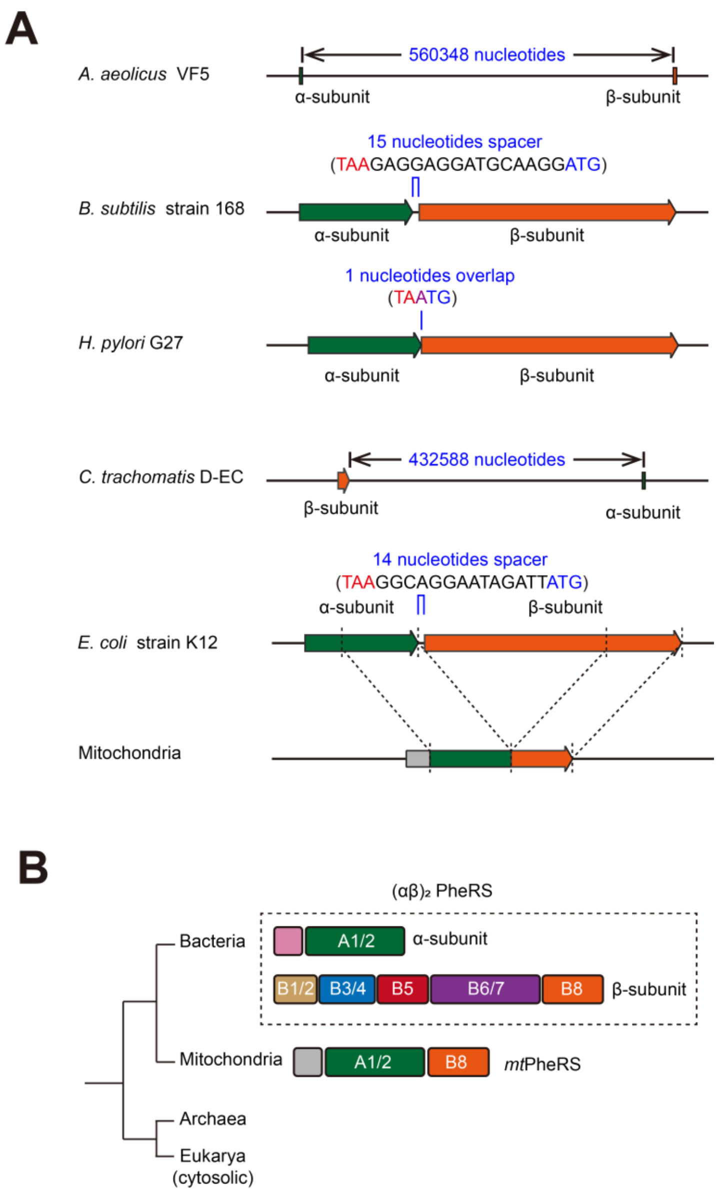


**Supplementary Figure S17. A diagram comparing human mtPheRS with the prokaryotic PheRS.** (**A**) Diagrammatic representation of the arrangements of the phenylalanyl-tRNA synthetase (PheRS) encoding genes found in the genomes of *Aquifex aeolicus* VF5 (GenBank: AE000657.1), *Bacillus subtilis* strain 168 (GenBank: NC_000964.3), *Helicobacter pylori* G27 (GenBank: CP001173.1), *Chlamydia trachomatis* D-EC (GenBank: CP002052.1). *Escherichia coli* strain K-12 (GenBank: CP009685.1) and *Homo sapiens* (chromosome 6, NCBI Reference Sequence: NG_033003.2). For the mitochondrial PheRS (*mt*PheRS), the regions similar to α- and β-subunits of the bacterial PheRS are indicated by the black dashed lines, and the N-terminal region of *mt*PheRS is colored gray. (**B**) Schematic representation of α- and β-subunits of the bacterial PheRS and mitochondrial PheRS in terms of structural domains.

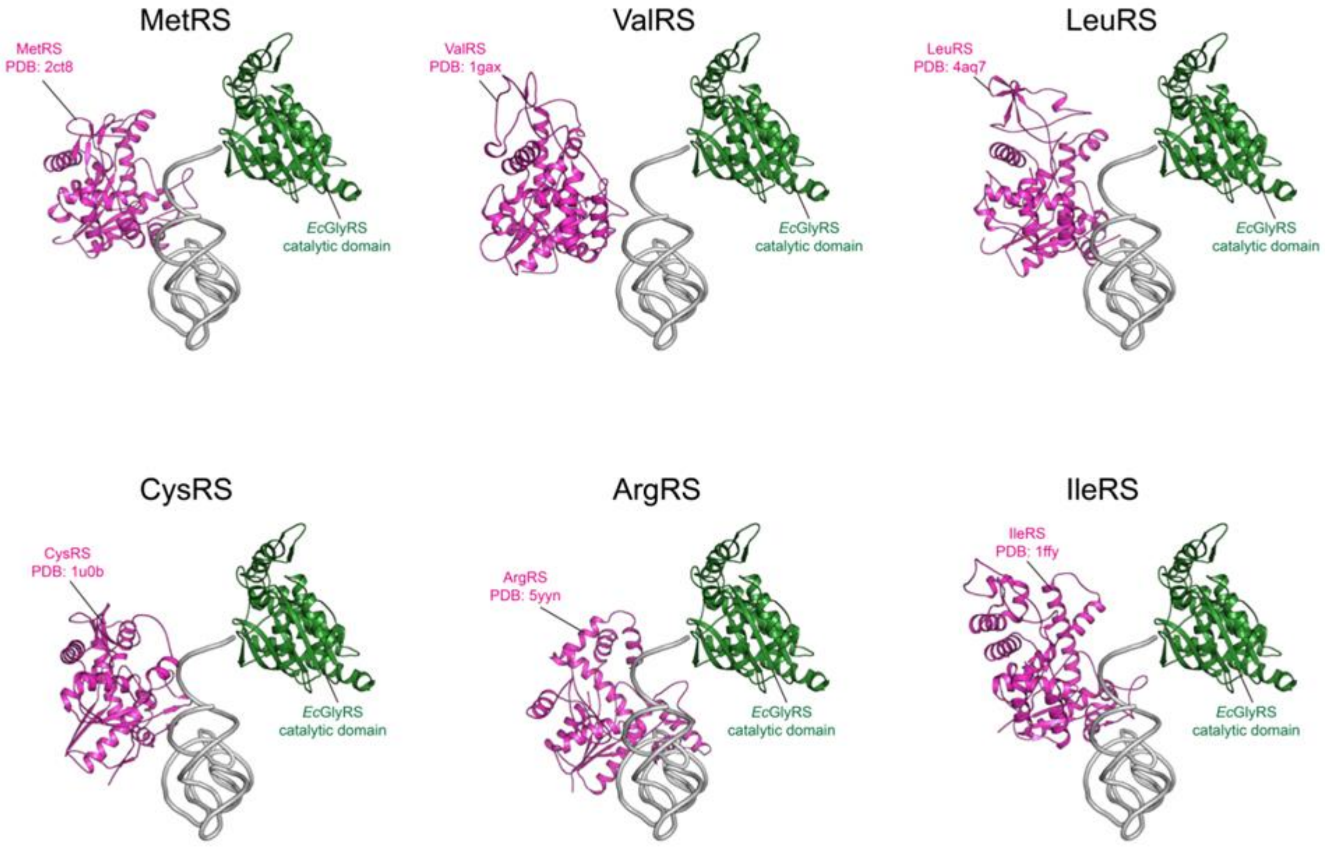


**Supplementary Figure S18. Modeled complexes suggested that *Ec*GlyRS α-subunit and the catalytic domains of class Ia synthetases could bind to tRNA acceptor stem simultaneously.** The modeled *Ec*GlyRS-tRNA$^{Gly}$ complex was superimposed to the co-crystal structures of class Ia synthetases in complex with their tRNAs by aligning the acceptor stems of the tRNA molecules. For clarity, only the tRNA$^{Gly}$ (gray), the α-subunit of *Ec*GlyRS (green) and the catalytic domains of class Ia aaRSs (magenta) are shown as cartoon. All the docking results are shown in two different orientations. The views show the molecules along the axis of the anticodon stem-loop, from the acceptor stem side.

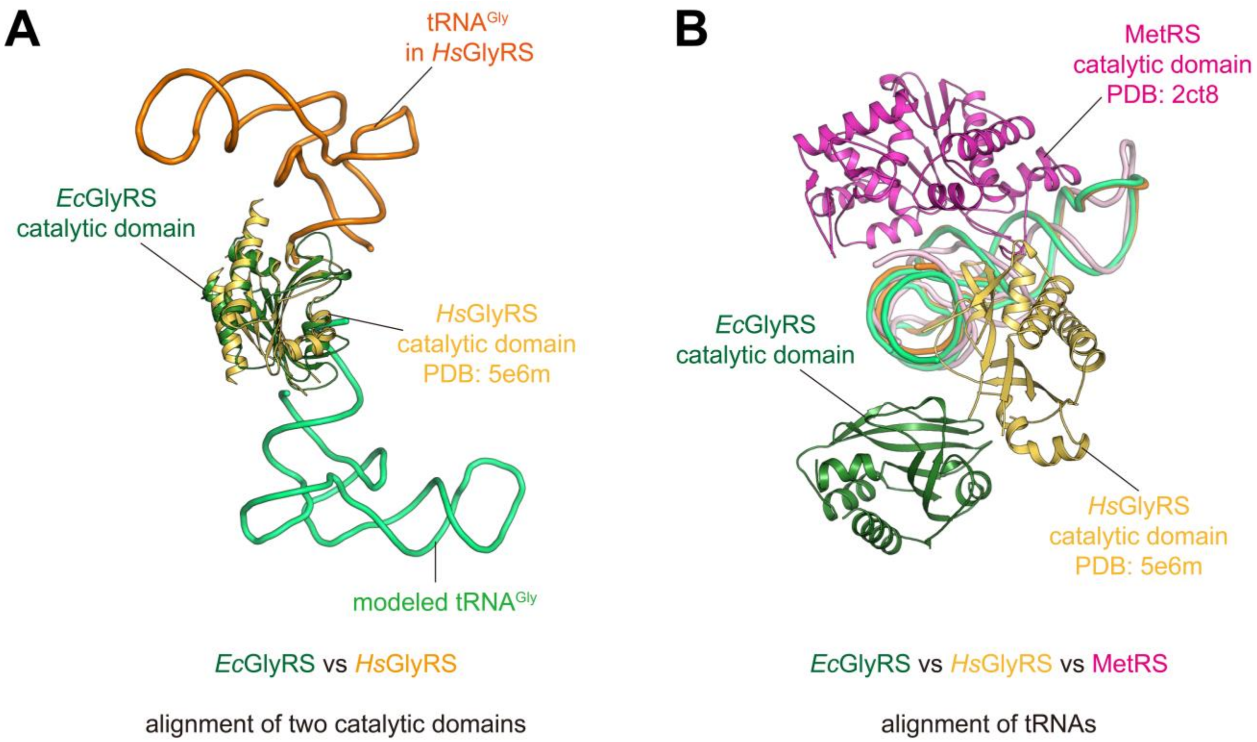


**Supplementary Figure S19. Structure superposition of the *Ec*GlyRS and *Hs*GlyRS.** (**A**) When catalytic domains of *Ec*GlyRS (green) and *Hs*GlyRS (yellow) were superimposed, the tRNA$^{Gly}$ molecules were found to bind to opposite sides of the catalytic domains of two GlyRSs. (**B**) When tRNA$^{Gly}$ molecules were aligned, the catalytic domains of *Ec*GlyRS (green) and *Hs*GlyRS (yellow) were found to bind the acceptor stem of tRNA$^{Gly}$ from different directions. Notably, the class Ia MetRS (magenta) could dock to acceptor stem of tRNA without clash with both types of GlyRS.

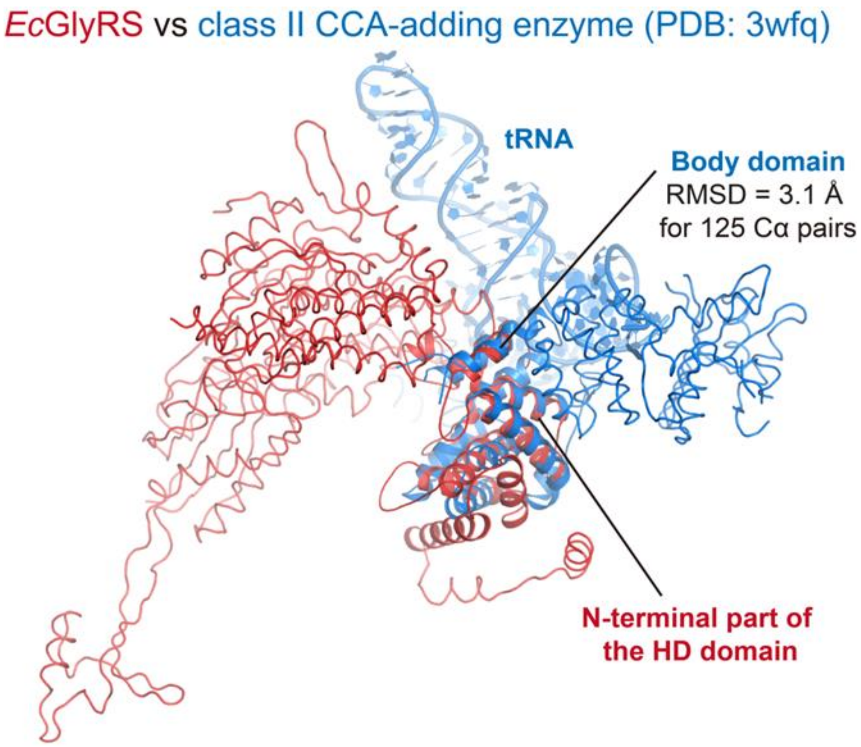


**Supplementary Figure S20. Structural superposition of the N-terminal part of the HD domain and the class II CCA-adding enzyme.** In *Aquifex aeolicus*, the CCA-3' is synthesized by CC-adding and A-adding enzymes in a collaborative manner, which are all belong to the class II CCA-adding enzymes. The body domain (shown as blue cartoon) of the *Aquifex aeolicus* CC-adding enzyme (PDB: 3wfq) is composed of a bundle of a-helices, which is involved in selecting and fixing the tRNA molecule onto the enzymes. The N-terminal part of the HD domain exhibits significant similarity to the body domain of class II CCA-adding enzymes.

**Supplementary Table 1. Statistics of X-ray diffraction data collection and structure refinement.**

| | |
|---|---|
| **Data collection** | |
| Resolution (Å) | 50.00-2.68 (2.78-2.68)[a] |
| Wavelength (Å) | 0.979 |
| Space group | *F*222 |
| Cell parameters | |
| *a*, *b*, *c* (Å) | *a*=207.4, *b*=253.9, *c*=270.7 |
| *α*, *β*, *γ* (°) | *α*=90.0, *β*=90.0, *γ*=90.0 |
| Unique reflections | 99458 (9862) |
| Redundancy | 13.5 (13.1) |
| Completeness (%) | 99.6 (92.9) |
| Average I/σ (I) | 42.5 (3.5) |
| $R_{merge}$[b] (%) | 8.4 (60.2) |
| **Refinement** | |
| Resolution (Å) | 48.58-2.68 (2.78-2.68) |
| Reflections for refinement/test | 94460/4996 |
| $R_{work}$[c]/$R_{free}$[d] (%) | 22.8/24.9 |
| RMSD bond (Å) | 0.003 |
| RMSD angle (°) | 1.21 |
| Mean B factor (Å$^2$) | 68.4 |
| Non-hydrogen protein atoms | 13275 |
| Water oxygen atoms | 56 |
| Other non-hydrogen atoms | 76 |
| MolProbity Ramachandran plot (%) | |
| Most favored regions | 96.4 |
| Additional allowed regions | 3.1 |
| Outliers | 0.5 |

[a]Values in parentheses are for the highest resolution shell.

[b]$R_{merge} = \Sigma_h \Sigma_l | I(h)_l - \langle I(h)\rangle | / \Sigma_h \Sigma_l I(h)_l$, where $I(h)_l$ is the *l*th observation of the reflection h and $\langle I(h)\rangle$ is the weighted average intensity for all observations *l* of reflection h.

[c]$R_{work}=\sum_h | | F_{obs}(h) | - | F_{cal}(h) | | /\sum_h | F_{obs}(h) |$, where $F_{obs}(h)$ and $F_{cal}(h)$ are the observed and calculated structure factors for reflection h respectively.

[d]$R_{free}$ was calculated as $R_{work}$ using 5.0% of the reflections which were selected randomly and omitted from refinement.

**Supplementary Table 2. The DALI result for the α-subunit in *Ec*GlyRS.**

| No: | Chain | Z | rmsd | lali | nres | %id | Description |
|---|---|---|---|---|---|---|---|
| 1: | 3rgl-A | 40.3 | 1.3 | 286 | 293 | 64 | Glycyl-tRNA synthetase alpha subunit; |
| 2: | 3wqy-A | 18.7 | 3.7 | 209 | 906 | 17 | Alanine--tRNA synthetase; |
| 3: | 3hxu-A | 16.7 | 3.0 | 195 | 442 | 14 | Alanyl-tRNA synthetase; |
| 4: | 2du7-A | 14.6 | 2.9 | 167 | 539 | 17 | O-phosphoseryl-tRNA synthetase; |
| 5: | 3reu-B | 14.6 | 2.7 | 172 | 290 | 13 | Asns-like asparaginyl-tRNA synthetase |
| 6: | 2odr-A | 14.1 | 2.7 | 162 | 491 | 18 | Phosphoseryl-tRNA synthetase; |
| 7: | 1eqr-A | 13.9 | 4.0 | 187 | 590 | 13 | Aspartyl-tRNA synthetase; |
| 8: | 6aqg-C | 13.8 | 2.7 | 165 | 491 | 13 | Lysine--tRNA synthetase; |
| 9: | 3ica-B | 13.7 | 2.4 | 144 | 207 | 8 | Phenylalanyl-tRNA synthetase beta chain; |
| 10: | 3dsq-B | 13.7 | 3.0 | 163 | 282 | 13 | Pyrrolysyl-tRNA synthetase; |
| 11: | 4up8-A | 13.7 | 2.9 | 168 | 580 | 14 | Lysine--tRNA synthetase; |
| 12: | 5mgu-A | 13.5 | 3.5 | 172 | 408 | 14 | Phenylalanine--tRNA synthetase, mitochondrial; |
| 13: | 6r02-C | 13.4 | 3.0 | 155 | 380 | 10 | ATP phosphoribosyltransferase regulatory subunit; |
| 14: | 1g5h-D | 13.1 | 3.6 | 154 | 415 | 7 | Mitochondrial DNA polymerase accessory subunit; |
| 15: | 4o2d-B | 13.1 | 3.6 | 180 | 516 | 14 | Aspartate--tRNA synthetase; |
| 16: | 3pco-B | 12.9 | 2.9 | 151 | 795 | 10 | Phenylalanyl-tRNA synthetase, beta subunit; |
| 17: | 1kmm-B | 12.9 | 2.8 | 162 | 365 | 15 | Histidyl-tRNA synthetase; |
| 18: | 11as-A | 12.7 | 2.9 | 169 | 328 | 11 | Asparagine synthetase; |
| 19: | 2zt7-A | 12.3 | 3.2 | 159 | 531 | 13 | Glycyl-tRNA synthetase; |
| 20: | 1adj-A | 12.3 | 2.8 | 156 | 421 | 17 | Histidyl-tRNA synthetase; |
| 21: | 4kqe-A | 12.3 | 3.1 | 155 | 597 | 14 | Glycine--tRNA synthetase; |
| 22: | 4yrn-A | 12.3 | 3.0 | 159 | 416 | 10 | Histidyl-tRNA synthetase; |
| 23: | 3l4g-C | 12.1 | 2.8 | 163 | 509 | 14 | Phenylalanyl-tRNA synthetase alpha subunit; |
| 24: | 5e6m-A | 12.0 | 3.5 | 156 | 520 | 13 | Glycine--tRNA synthetase; |
| 25: | 6pqh-B | 12.0 | 2.7 | 168 | 479 | 12 | Asparagine--tRNA synthetase; |
| 26: | 1z7m-B | 11.9 | 2.8 | 155 | 318 | 12 | ATP phosphoribosyltransferase regulatory subunit; |
| 27: | 3w3s-A | 11.4 | 3.2 | 164 | 527 | 12 | Serine--tRNA synthetase; |
| 28: | 3mf2-A | 11.3 | 3.7 | 155 | 299 | 17 | BLL0957 protein; |
| 29: | 6od8-A | 11.1 | 2.7 | 170 | 506 | 9 | Putative aspartyl-tRNA synthetase; |
| 30: | 1nyq-B | 11.1 | 3.2 | 160 | 646 | 11 | Threonyl-tRNA synthetase; |

**Supplementary Table 3. The DALI result for the B1-B2 domain from β-subunit in *Ec*GlyRS.**

| No: | Chain | Z | rmsd | lali | nres | %id | Description |
|---|---|---|---|---|---|---|---|
| 1: | 3ovs-B | 6.9 | 4.0 | 113 | 437 | 10 | Transposase, putative; |
| 2: | 2fyx-B | 6.5 | 3.6 | 95 | 130 | 12 | CCA-adding enzyme; |
| 3: | 2zh6-A | 6.4 | 4.3 | 114 | 437 | 10 | CCA-adding enzyme; |
| 4: | 1tfy-B | 6.4 | 4.1 | 112 | 437 | 10 | CCA-adding enzyme; |
| 5: | 2dr7-A | 6.4 | 4.1 | 113 | 437 | 10 | CCA-adding enzyme; |
| 6: | 2zh1-A | 6.4 | 4.0 | 113 | 437 | 10 | CCA-adding enzyme; |
| 7: | 1tfw-A | 6.4 | 4.0 | 111 | 437 | 10 | Transposase, putative; |
| 8: | 2f4f-B | 6.4 | 2.6 | 91 | 130 | 9 | CCA-adding enzyme; |
| 9: | 1r8b-A | 6.4 | 4.3 | 120 | 437 | 10 | CCA-adding enzyme; |
| 10: | 1r89-A | 6.3 | 4.3 | 112 | 437 | 10 | CCA-adding enzyme; |
| 11: | 2zh9-A | 6.3 | 4.1 | 113 | 437 | 10 | CCA-adding enzyme; |
| 12: | 1uev-A | 6.3 | 4.1 | 112 | 431 | 10 | CCA-adding enzyme; |
| 13: | 2drb-A | 6.3 | 4.1 | 113 | 437 | 10 | CCA-adding enzyme; |
| 14: | 2dvi-A | 6.3 | 4.2 | 114 | 431 | 10 | CCA-adding enzyme; |
| 15: | 1r8a-A | 6.3 | 4.2 | 112 | 437 | 10 | CCA-adding enzyme; |
| 16: | 1tfw-D | 6.3 | 4.1 | 111 | 437 | 10 | CCA-adding enzyme; |
| 17: | 2zh7-A | 6.3 | 4.1 | 113 | 436 | 10 | CCA-adding enzyme; |
| 18: | 1sz1-A | 6.3 | 4.1 | 112 | 437 | 10 | CCA-adding enzyme; |
| 19: | 2zh2-A | 6.3 | 4.0 | 112 | 437 | 10 | CCA-adding enzyme; |
| 20: | 4x4n-A | 6.3 | 4.2 | 112 | 436 | 10 | CCA-adding enzyme; |
| 21: | 1tfy-D | 6.3 | 4.4 | 113 | 437 | 10 | CCA-adding enzyme; |
| 22: | 1r8c-A | 6.3 | 4.2 | 113 | 437 | 10 | CCA-adding enzyme; |
| 23: | 1tfw-B | 6.3 | 4.0 | 111 | 437 | 10 | CCA-adding enzyme; |
| 24: | 1tfw-C | 6.3 | 4.1 | 113 | 437 | 10 | CCA-adding enzyme; |
| 25: | 2zhb-A | 6.3 | 4.0 | 113 | 436 | 10 | Transposase, putative; |
| 26: | 2f5g-B | 6.3 | 2.5 | 88 | 130 | 9 | Transposase; |
| 27: | 2xo6-A | 6.3 | 3.0 | 87 | 134 | 8 | CCA-adding enzyme; |
| 28: | 3ovb-B | 6.3 | 4.1 | 115 | 437 | 10 | CCA-adding enzyme; |
| 29: | 2hvh-A | 6.3 | 2.6 | 90 | 580 | 12 | DNA polymerase; |
| 30: | 4yfu-A | 6.3 | 2.7 | 90 | 579 | 12 | Transposase, putative; |

**Supplementary Table 4. The DALI result for the HD domain from β-subunit in *Ec*GlyRS.**

| No: | Chain | Z | rmsd | lali | nres | %id | Description |
|---|---|---|---|---|---|---|---|
| 1: | 3mem-A | 11.6 | 3.0 | 139 | 453 | 19 | Putative signal transduction protein; |
| 2: | 2pq7-A | 11.3 | 3.1 | 132 | 174 | 17 | Predicted HD superfamily hydrolase; |
| 3: | 4s1b-D | 11.1 | 3.0 | 142 | 214 | 9 | LMO1466 protein; |
| 4: | 2o08-A | 10.3 | 3.4 | 118 | 187 | 17 | BH1327 protein; |
| 5: | 3hc1-A | 10.1 | 2.8 | 136 | 298 | 17 | Uncharacterized HDOD domain protein; |
| 6: | 3ccg-A | 10.1 | 4.3 | 121 | 188 | 21 | HD superfamily hydrolase; |
| 7: | 3nqw-A | 9.8 | 3.4 | 144 | 178 | 13 | CG11900; |
| 8: | 2cqz-A | 9.8 | 3.2 | 134 | 173 | 17 | 177 aa long hypothetical protein; |
| 9: | 4mcw-B | 9.6 | 3.2 | 137 | 363 | 12 | Metal dependent phosphohydrolase; |
| 10: | 3mzo-B | 9.6 | 2.9 | 138 | 211 | 14 | LIN2634 protein; |
| 11: | 3m1t-A | 9.5 | 3.1 | 133 | 269 | 11 | Putative phosphohydrolase; |
| 12: | 2par-B | 9.3 | 3.4 | 134 | 178 | 10 | 5'-deoxynucleotidase YFBR; |
| 13: | 2ogi-B | 9.0 | 3.6 | 119 | 194 | 14 | Hypothetical protein SAG1661; |
| 14: | 3p3q-B | 9.0 | 3.2 | 129 | 253 | 12 | MMOP; |
| 15: | 4mlm-A | 8.8 | 3.7 | 119 | 188 | 14 | Predicted HD phosphohydrolase phnz; |
| 16: | 4r8z-A | 8.8 | 3.2 | 136 | 218 | 13 | Cyclic di-GMP phosphodiesterase; |
| 17: | 3gw7-A | 8.6 | 4.2 | 127 | 215 | 14 | Uncharacterized protein YEDJ; |
| 18: | 2pjq-B | 8.6 | 3.9 | 138 | 212 | 17 | Uncharacterized protein LP_2664; |
| 19: | 6s2t-A | 8.5 | 4.7 | 147 | 350 | 9 | (P)PPGPP synthetase I, Spot/RelA; |
| 20: | 6pbz-A | 8.5 | 4.1 | 125 | 485 | 14 | Guanosine-5'-triphosphate,3'-diphosphate pyrophos |
| 21: | 6npa-A | 8.4 | 3.6 | 114 | 188 | 8 | TMPB, (R)-1-hydroxy-2-trimethylaminoethylphosphon |
| 22: | 3tmb-B | 8.3 | 3.2 | 129 | 318 | 13 | Uncharacterized protein BD1817; |
| 23: | 1u6z-B | 8.2 | 4.1 | 126 | 500 | 7 | Exopolyphosphatase; |
| 24: | 3i7a-A | 8.2 | 3.3 | 137 | 280 | 12 | Putative metal-dependent phosphohydrolase; |
| 25: | 3wfr-H | 8.1 | 3.3 | 126 | 463 | 18 | Class II CCA-adding enzyme; |
| 26: | 5ihy-B | 8.0 | 3.6 | 129 | 193 | 16 | Uncharacterized protein; |
| 27: | 3b57-A | 8.0 | 3.4 | 117 | 180 | 12 | LIN1889 protein; |
| 28: | 2zze-A | 8.0 | 3.7 | 129 | 744 | 12 | Alanyl-tRNA synthetase; |
| 29: | 5tk6-A | 7.9 | 3.7 | 131 | 191 | 9 | OXSA protein; |
| 30: | 3hi0-A | 7.9 | 2.8 | 123 | 501 | 12 | Putative exopolyphosphatase. |

**Supplementary information reference**

1. Sievers, F., Wilm, A., Dineen, D., Gibson, T.J., Karplus, K., Li, W., Lopez, R., McWilliam, H., Remmert, M., Soding, J. *et al.* (2011) Fast, scalable generation of high-quality protein multiple sequence alignments using Clustal Omega. *Mol Syst Biol*, **7**, 539.
2. Waterhouse, A.M., Procter, J.B., Martin, D.M., Clamp, M. and Barton, G.J. (2009) Jalview Version 2--a multiple sequence alignment editor and analysis workbench. *Bioinformatics*, **25**, 1189-1191.
